# Estimating Structural Damage to Mangrove Forests Using Airborne Lidar Imagery: Case Study of Damage Induced by the 2017 Hurricane Irma to Mangroves in the Florida Everglades, USA

**DOI:** 10.3390/s23156669

**Published:** 2023-07-25

**Authors:** Selena Chavez, Shimon Wdowinski, David Lagomasino, Edward Castañeda-Moya, Temilola Fatoyinbo, Ryan P. Moyer, Joseph M. Smoak

**Affiliations:** 1 Institute of Environment, Department of Earth and Environment Florida International University, Miami, FL 33199, USA; swdowins@fiu.edu; 2 Integrated Coastal Programs, East Carolina University, Wanchese, NC 27981, USA; lagomasinod19@ecu.edu; 3 Institute of Environment, Department of Biological Sciences, Florida International University, Miami, FL 33199, USA; ecastane@fiu.edu; 4 Biospheric Sciences Laboratory, NASA Goddard Space Flight Center, Greenbelt, MD 20771, USA; lola.fatoyinbo@nasa.gov; 5 TerraCarbon LLC, Peoria, IL 61614, USA; ryan.moyer@terracarbon.com; 6 School of Geosciences, University of South Florida, St. Petersburg, FL 33701, USA

**Keywords:** lidar, mangrove, hurricane, South Florida, Everglades

## Abstract

In September 2017, Hurricane Irma made landfall in South Florida, causing a great deal of damage to mangrove forests along the southwest coast. A combination of hurricane strength winds and high storm surge across the area resulted in canopy defoliation, broken branches, and downed trees. Evaluating changes in mangrove forest structure is significant, as a loss or change in mangrove forest structure can lead to loss in the ecosystems services that they provide. In this study, we used lidar remote sensing technology and field data to assess damage to the South Florida mangrove forests from Hurricane Irma. Lidar data provided an opportunity to investigate changes in mangrove forests using 3D high-resolution data to assess hurricane-induced changes at different tree structure levels. Using lidar data in conjunction with field observations, we were able to model aboveground necromass (AGN; standing dead trees) on a regional scale across the Shark River and Harney River within Everglades National Park. AGN estimates were higher in the mouth and downstream section of Shark River and higher in the downstream section of the Harney River, with higher impact observed in Shark River. Mean AGN estimates were 46 Mg/ha in Shark River and 38 Mg/ha in Harney River and an average loss of 29% in biomass, showing a significant damage when compared to other areas impacted by Hurricane Irma and previous disturbances in our study region.

## 1. Introduction

Mangroves provide essential ecosystem services, including mitigation of large storms and flooding, coastline stabilization, habitat for many species (some vulnerable and endangered), essential nutrient cycling, and carbon storage and sequestration [1,2,3]. Globally it is estimated that mangroves provide flood protection from tropical storms and hurricanes at an estimated value of 65 billion USD per year, with the U.S.A. being one of the countries with the greatest economic benefit [3]. At a more local scale, it is estimated that mangroves in South Florida averted an estimated $1.5 billion USD in damages from Hurricane Irma in 2017, and mangroves in the Everglades provide an estimated economic value of $2–3 billion USD in potential carbon storage [1,4]. Over the last several decades mangroves have been rapidly disappearing at alarming rates, with 80% of global mangrove losses attributed to human impacts and 14% of global losses estimated to be caused by extreme weather events such as hurricanes [5].

Due to the geographic location in the sub-tropics, mangroves in South Florida are periodically impacted by tropical storms and hurricanes [6,7,8,9]. Mangroves in South Florida have been impacted by four major hurricanes over the past three decades (Hurricane Andrew in 1992, Hurricanes Katrina and Wilma in 2005, and Hurricane Irma in 2017). Such storms can cause significant damage and loss of mangroves, which decrease mangroves’ capability to provide ecosystem services [6]. Traditionally, to assess the effects of large disturbances, such as hurricanes on mangroves, field surveys can be conducted. This includes establishing vegetation plots and assessing the condition of mangroves post-disturbance. Field assessments can provide key information on the status and recovery trajectories of mangroves post-disturbance. However, field assessments can be both time-consuming and physically taxing due to the nature of maneuvering through wetland environments and the hazards of walking through mangrove prop roots. Field assessments also may only cover small areas and provide information at a small scale. To evaluate the impact of storm-induced disturbances on mangroves, we can use remote sensing to assess and quantify potential damage at regional spatial scales.

Remotely sensed data captured from numerous different sensors have successfully been used in past studies to assess hurricane-induced mangrove damage in South Florida. In 2018, time series (1985 to 2017) analysis from Landsat, an optical remote sensing satellite, was used to detect changes from four different major hurricanes (Hurricane Andrew in 1992, Hurricanes Katrina and Wilma in 2005, and Hurricane Irma in 2017)) that impacted mangroves in the Everglades. Their results indicated a significant decrease in mangrove cover, with a post-disturbance recovery of 3–4 years [10]. A similar study in 2012 used MODIS, a remote sensing instrument aboard the Terra and Aqua satellites, to look at the effects of Hurricane Katrina and Hurricane Wilma on mangroves of the Everglades. Using the Normalized Difference Vegetation Index (NDVI), they found that post-storm a large canopy decrease can be detected, and it takes 2–3 years for this canopy to recover, with the fastest recovery occurring within the first year [11]. Airborne lidar observations used after Hurricane Wilma in 2005 were able to detect large mangrove defoliation within the Everglades, which led to the creation of large gaps within the canopy and increased amounts of lower canopy and ground cover observations [12]. The damage induced by Hurricanes Andrew, Katrina, and Wilma was also studied using NDVI change analysis derived from Landsat imagery and airborne lidar. These NDVI analyses and lidar data revealed a negative relationship between the distance of mangroves from a hurricane eyewall and canopy loss, which was confirmed by field data [12]. Lidar and other optical imagery analyses suggested Hurricane Irma caused a record-breaking loss of 10,769 ha of mangroves within South Florida, which was mostly attributed to poor drainage and storm surge ponding, and an estimated 15.3% loss in canopy volume [8]. Hurricane Irma also caused canopy damages to an estimated 62% of mangrove forests in the Everglades, with tall trees (>10 m tall) being most affected and storm winds causing significant mangrove canopy defoliation and tree snapping and uprooting [8]. A recent study using lidar also confirmed that the majority of the damage from Hurricane Irma was concentrated in tall mangroves (15–25 m tall) and that shorter mangroves (<5 m) are more resilient to storm effects and experience the least amount of damage [9]. This study also found that only 38.1% of mangroves that experienced canopy loss from Hurricane Irma had recovered to pre-storm canopy height almost 2.5 years later, which provides further evidence that hurricanes significantly alter mangrove canopy structure and overall height distribution of mangroves within the study region post-hurricane [9]. These studies prove that remote sensing is a powerful tool in being able to detect large-scale effects of hurricane disturbance on mangroves, but the majority of these studies have only been able to quantify changes to mangroves from a canopy level. No available study has been able to detect volumes of damage in terms of aboveground necromass (AGN). Modeling and estimating volumes of AGN would allow us to better understand how hurricanes affect mangrove tree structure and not just the canopy as documented in previous studies [8,9,10,11].

In this study, we use airborne lidar data obtained from NASA’s Goddard’s Lidar, Hyperspectral & Thermal Imager (G-LiHT) and field data to estimate hurricane-induced structural damages of mangrove forests in the Florida Everglades following the passage of Hurricane Irma on 10 September 2017 across the region. G-LiHT has been successfully used in past studies in both South Florida and Puerto Rico in order to detect changes in mangroves after a hurricane disturbance, making it a very useful tool [8,9,13]. Our main objective is to quantify volumes of damage and analyze the distribution of damage (i.e., AGN) to the mangroves of the Everglades following Hurricane Irma. We addressed the following questions: (1) Can we quantify volumes of woody debris (WD) and AGN to assess mangrove damage after hurricane disturbance using both lidar and field data? (2) Can we detect a local and landscape pattern of hurricane damage based on volume estimates of WD and AGN? Using field measurements of WD and AGN and metrics obtained from airborne lidar data, we analyzed relationship patterns between the two data sets to upscale volumes of WD and AGN over larger areas and assessed patterns of distribution. Our study presents the first attempt to quantify and upscale volumes of WD and AGN regionally using airborne observations.

## 2. Background

### 2.1. Mangroves of the Everglades National Park

**Figure 1:**
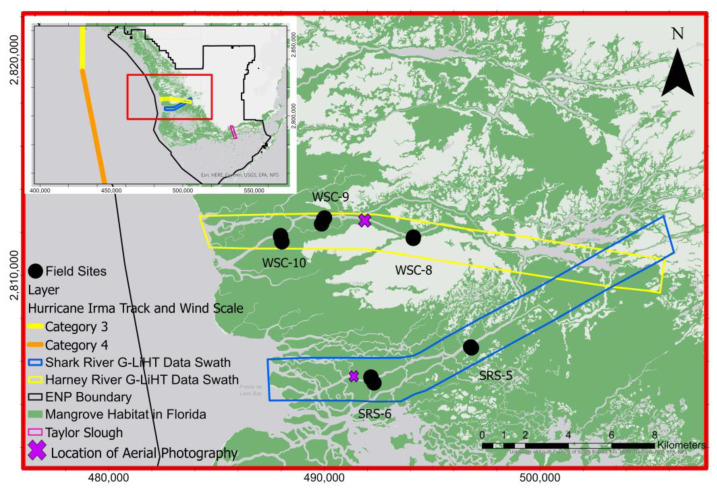
Map of mangrove forests in Everglades National Park, South Florida, showing the locations of 13 field plots in the southwestern Everglades, G-LiHT, data swath, the proximity of Hurricane Irma’s track through South Florida, and location of aerial photography.

Mangrove forests located in the Everglades National Park (from here on referred to as the Everglades) are the most extensive mangrove area in continental North America. They are located mainly along the Gulf of Mexico, covering an estimated area of 144,000 ha [14]. Our study region in the Everglades consists of two east-west swathes located along two of the major rivers in the southwestern Everglades, Shark River and Harney River (Figure 1). These two rivers are significant distributaries of the greater Everglades ecosystem and are mostly surrounded by mangrove forests. Mangrove forests within the Everglades contain mixed species of red mangroves (*Rhizophora mangle*), white mangroves (*Laguncularia racemosa*), and black mangroves (*Avicennia germinans*). The buttonwood mangrove species (*Conocarpus erectus*) is restricted to upstream locations of estuaries (e.g., Shark River) in the southwestern Everglades [15]. Some of the most productive and structurally developed mangroves are found in the Shark River area, particularly near the mouth of the estuary, with aboveground biomass (AGB) estimates ranging from 95 to 162 Mg/ha and some areas estimated to be as high as 250 Mg/ha [15,16]. Before Hurricane Irma impacted these mangroves, their average height reached 12–18 m [14]. South Florida is considered a tropical climate, with a wet season from May to October and a Dry Season from November to April, where most of the rainfall (60%) and hurricane development occur during the wet season [17]. Mangrove areas within the Everglades boundary are part of the Florida Coastal Everglades Long-Term Ecological Research (FCE-LTER) program (https://fcelter.fiu.edu/, (accessed on 1 December 2022)).

### 2.2. The 2017 Hurricane Irma

**Figure 2:**
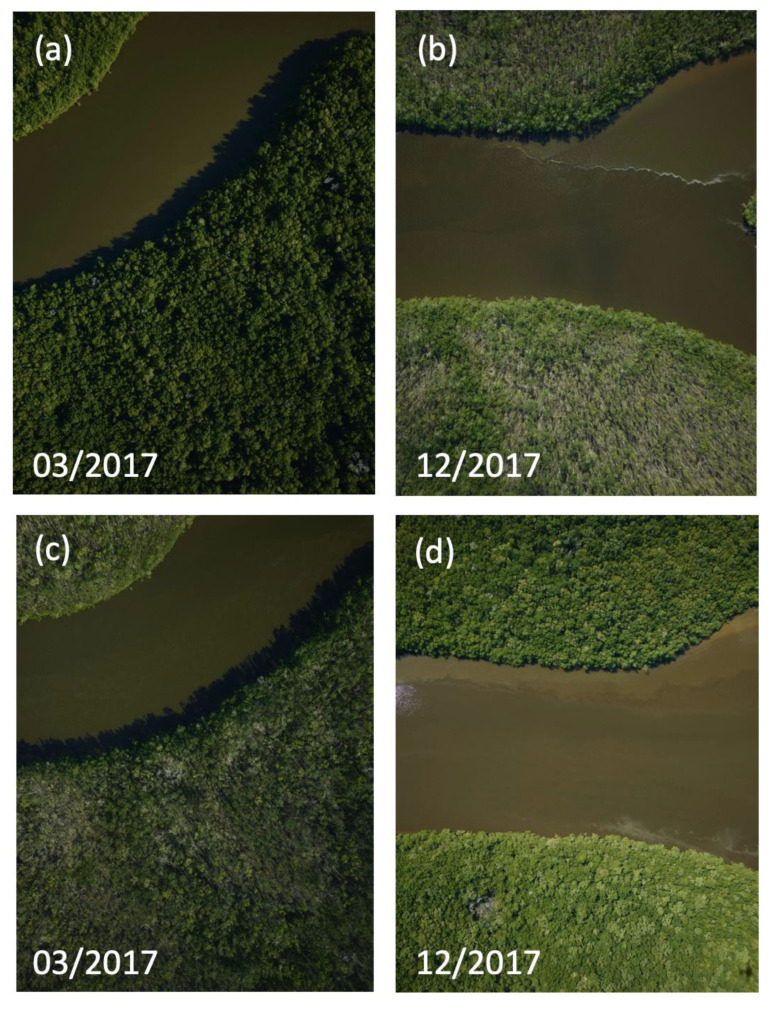
High-resolution imagery obtained from NASA G-LiHT shows evidence of damage induced by Hurricane Irma. Images (**a**,**c**) show the mangrove canopy before Hurricane Irma and images (**b**,**d**) display the mangrove canopy after Irma. The locations of the images are shown in Figure 1.

Hurricane Irma made landfall as a Category 3 storm on mainland Florida (Figure 1) on 10 September 2017, with an estimated wind speed of 112 to 120 mph [18]. Inundation levels of 1.83–3.05 m were reported within the Everglades [18]. The storm surge produced maximum water levels up to 3 m above ground level along the southwest coast of Florida [18]. Water levels within mangrove forests increased as a result of the storm surge, with the highest near the mouth of the Shark River estuary and the lowest at upstream locations. Water levels peaked at about 1 m above the soil surface at SRS-6 (near the mouth) and ~0.75 m at mid- and upstream locations (SRS-5) of the Shark River estuary. Similar trends in water levels were observed in the Harney River [7]. The estimated rainfall was 10–15 inches (25.3–38.1 cm), as measured in most of the state of Florida [18]. Due to its strength, Hurricane Irma caused widespread damage to many areas in South Florida. Mangrove forests along the Shark and Harney Rivers were located along the hurricane’s eye path (Figure 1) and, consequently, experienced heavy damage, particularly in near-coast mangrove areas, including defoliation, branch snapping, and uprooting of mangrove trees. Evidence of mangrove damage is well observed in the high-resolution aerial photography acquired before and after the hurricane (Figure 2). All high-resolution aerial imagery from G-LiHT is available at https://gliht.gsfc.nasa.gov/ (accessed on 1 March 2022). Prior to Hurricane Irma, the mangrove forest canopy along both the Shark and Harney Rivers was characterized by dense closed green crowns of trees with no visible damage. In contrast, after Hurricane Irma impacted the area, G-LiHT images revealed several brown spots due to canopy gaps created by the storm, reflecting tree defoliation and snapping (Figure 2). 

### 2.3. NASA’s G-LiHT

NASA’s new generation G-LiHT is an airborne imaging system that maps the composition, structure, and function of terrestrial ecosystems. The G-LiHT system includes a lidar scanner, which provides 3D information on foliage and canopy elements using a pulsed laser, and hyperspectral and thermal sensors [19]. The G-LiHT imager has a 387-m-wide swath at a flight altitude of 335 m above the surface and, hence, produces data at a fine spatial resolution of less than 1 m. 

Lidar data from G-LiHT surveys are available at the NASA GSFC archive (https://glihtdata.gsfc.nasa.gov/ (accessed on 1 March 2022)) in LASer (LAS) format files. G-LiHT LAS files include the X, Y, and Z coordinate positions of each lidar pulse return (point clouds), which are georeferenced by an onboard GPS and Inertial Navigation System (INS). The G-LiHT archive also includes gridded higher-level products based on developed metrics with a resolution of 13 m for most metrics, including the fraction of first returns intercepted by trees, mean tree height, canopy height models, and mean ground returns; these data products are available as GeoTIFFs files, which store georeferenced data. The complete list of G-LiHT metric products is available at https://glihtdata.gsfc.nasa.gov/misc/metrics_readme.pdf (accessed on 1 March 2022).

## 3. Data

This study utilizes point cloud lidar data acquired by G-LiHT and field measurements of WD and AGN collected in both the Shark and Harney Rivers. The two datasets complement one another, as the high-spatial-resolution G-LiHT data can be used to upscale field data.

### 3.1. Lidar Datasets

Lidar point cloud data were acquired by the G-LiHT sensor in March and December 2017. The March 2017 acquisition took place six months before Hurricane Irma impacted the study region, and the December 2017 acquisition took place three months after the hurricane. The data were collected along seven parallel 387-m-wide swathes, covering a 1300 m wide swath along both the Shark and Harney Rivers (Figure 1). For both the March 2017 and December 2017 flights, the same instruments (known as G-LiHT v2.0) and the same flight path were taken for both data acquisitions. Data from G-LiHT are comprised of large LAS files that cover our study region along the Shark River and Harney River. In this study, we use both the raw point clouds, which were acquired with spatial resolution of less than 1 m, and higher-level data products, which are available at 13 m spatial resolution. The gridded products also referred to as metrics, which take the raw lidar point cloud and scale data into 13 m pixels, used in this study and their definitions are listed in Table 1.

### 3.2. Field Measurements 

In January 2018, we conducted a spatially explicit sampling design along both the Shark River and Harney River to assess the immediate impact of Hurricane Irma on mangrove forest structure from upstream to downstream locations along each estuary [20]. Field measurements were conducted within 10 × 10 m vegetation plots that were established along six transects perpendicular to the mangrove shoreline at each site, at 50, 100, and 350 m. The Shark River transects were established at 0, 4 (SRS-6), and 9 (SRS-5) km from the river’s mouth. However, we used only data from the 4- and 9-km transects, because the Shark River’s mouth (0 km transect) was not covered by the G-LiHT surveys (Figure 1). The Harney River transects were established at 2 (WSC-10), 6 (WSC-9), and 10 km (WSC-8) from the river’s mouth (Figure 1). Out of the fifteen measured field plots, we used thirteen field plots in this study: five field plots in the Shark River and eight field plots in the Harney River. Only thirteen out of the fifteen plots were used due to two field plots being located outside the G-LiHT data collection swath over the Shark River.

## 4. Methods

Our methodology relied on a five-stage procedure that used both lidar and field data. The first stage included lidar data extraction and processing lidar data to be clipped to the location of the 13 field plots. The second stage used field observations to estimate WD and AGN in the 13 plots. The third stage used both the field-based estimates of WD and AGN and lidar data to run linear regression analyses to model estimates of WD and AGN. We also used the lidar’s canopy height model to estimate AGT. The fourth stage used resampling statistical methods to calculate the uncertainty levels of the lidar-based estimates of WD and AGN. The fourth stage is upscaling, in which WD and AGN were calculated for the entire study area along the Shark and Harney Rivers.

### 4.1. Lidar Data Extraction

The G-LiHT data were extracted from large LAS files using the lidR software package, which is an R studio software package created to read and process airborne lidar [21]. The extracted data were formatted as 3D point clouds for each vegetation plot from before and after Irma. From there, lidar data were clipped to each of the 13 field plots, which were calculated using our selected metrics based on the original gridded data provided by G-LiHT. The selected metrics followed the standard G-LiHT metrics (Table 1) and included F-Cover, Tree Mean, Shrub Mean, and Pulse Density. Shrub Mean is used to describe average lidar data below a height of 1.37 m and should not be used to denote scrub mangroves (tree height < 2 m). These mangrove ecotypes are not present within the Shark and Harney Rivers [22]. G-LiHT metrics were calculated for each of the 13 plots before and after Hurricane Irma. The difference and percent change for each of the metrics before and after Irma were then calculated for each of the 13 field plots. 

### 4.2. Field-Based Estimates of AG, WD, and AGTM

We used the line-intercept technique originally proposed by Van Wagner (1968) and Brown (1974) and later applied to mangrove forests to evaluate the spatial variation in woody debris (WD) [23,24,25]. At each plot, five 10 m transects were randomly established from the center of the plot and treated as replicates. Woody debris was measured at 1 m intervals along transects and coarse (≥7.5 cm in diameter) and fine (<7.5 cm in diameter) WD intersecting the line along the 10 m transect were measured to 0.1 cm with a DBH measurement tape. WD represents the sum of coarse and fine values.

Within each plot, all trees (i.e., standing live and dead trees) with a diameter at breast height (DBH, 1.3 m) ≥ 5 cm were measured to determine species composition and tree density. Aboveground biomass (AGB; standing live trees) and necromass (AGN; standing dead trees) were calculated for each individual tree measured within each field plot using mangrove species-specific allometric equations published for the study region [26]. Aboveground total mass (AGTM), which is AGB + AGN, was also calculated for each field plot. The allometric equation used to calculate total mass for each species of mangrove tree was as follows:

(1) $$Total\;mass=(\log_{10}y=a\times \log_{10}\left(DBH\right)+b)-(\log_{10}y=c\times \log_{10}\left(DBH\right)$$

where *y* is mass in kg and Diameter at Breast Height (*DBH*) is in cm; *a* = 1.934, *b* = −0.395, *c* = 0.985, and *d* = −0.855 for black mangroves; *a* = 1.930, *b* =−0.441, *c* = 1.160, and *d* = −1.043 for white mangroves; and *a* =1.731, *b* = −0.112, *c* = 1.337, and *d* = −0.843 for red mangroves.

### 4.3. AGN and WD Regression Models and Modeled AGTM

As our regression analysis and validation studies (stages 2 and 3) are focused on small areas surrounding the 13 field plots, we extracted data for the specific plot locations from the large LAS files. Although the vegetation plots were 10 m × 10 m in size, we extracted data of larger areas and averaged the metric data using a 3 × 3 averaging spatial filter, because the positioning of the field plot was determined by hand-held GPS with roughly 3 m accuracies. Evaluating lidar data from a wider area can compensate for possible offset in the actual location of the field plot.

To use the G-LiHT observations to estimate structural damage induced by the 2017 Hurricane Irma on the mangrove forests in the Everglades, we used field measurements of AGN and WD to run regression analyses. To run a linear regression analysis, we used data between the estimated field measurements and a linear combination of G-LiHT data (3D point clouds) or processed G-LiHT metrics (Table 1). We explored a variety of regression models using the following metrics: F-Cover, Tree Mean, Shrub Mean, and Pulse Density, from before and after the hurricane. The fit of the regression models was determined by the regression coefficient (R^2^) and the *p*-value statistical test. Reliable models require an R^2^ greater than 0.5 and a *p*-value less than 0.05.

We also used the canopy height model (CHM) calculated from G-LiHT after Hurricane Irma to calculate AGTM for our study region to analyze what percentage of AGTM was made up of AGN post-storm. We used the allometric equation from Simard et al. in 2013 for AGB:

(2) B=10.0×H

where *B* is AGB in Mg/ha and *H* is height in meters to calculated AGTB within the mangrove of the Everglades [14]. Allometric equations for AGB can be used to model AGTM post-disturbance, as modeled values of “AGB” would include both live and dead standing trees post-disturbance if we are only using canopy height as are independent variable. 

### 4.4. Uncertainty Analysis 

The validation of the AGN and WD models is based on the Leave One Out Cross Validation (LOOCV) k-fold analysis, which can be used to estimate the error in a regression. LOOCV works by setting a training dataset and a test dataset for a model [27]. For each iteration the LOOCV is run, a data point is removed from the training set, and the LOOCV model tries to predict the excluded point and calculate errors from the training set. LOOCV iterations are run multiple times, removing a new data point each time and calculating a new error. Once all LOOCV iterations are run, then the average error for a model can be calculated to assess model estimations. We ran the LOOCV using the sklearn.model_selection cross-validation package tool within Python. 

### 4.5. Upscaling

We upscaled our calculated regression models by processing and mosaicking G-LiHT tiles of the provided G-liHT metrics (Table 1) that covered the entire swath of the Shark and Harney Rivers. The mosaiced tiles were clipped based on the National Park Service map of mangroves to run the model on an area with only mangrove forests [28]. A 3 × 3 spatial filter was then applied to get averaged values of each of the metrics used for the study region.

## 5. Results

### 5.1. Point Cloud Generation

**Figure 3:**
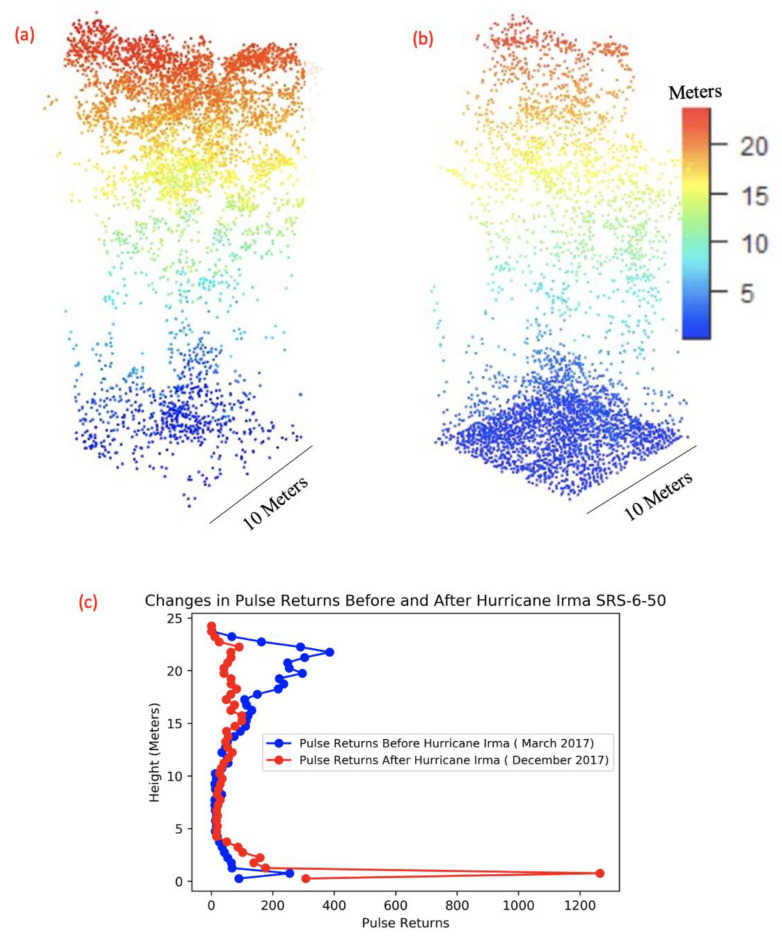
Point cloud variations of a single mangrove tree in plot SRS-6-50 in the Shark River before (**a**) and after (**b**) passage of Hurricane Irma. Distribution of pulse returns from point cloud data (**c**) at different height intervals from before Hurricane Irma versus after Hurricane Irma has been plotted. It can be noted that a high concentration of point returns is shown in higher elevation (canopy level) before Irma and at a lower elevation near soil surface after Irma.

We calculated 3D point clouds from the March and December 2017 datasets for 13 field 10 × 10 m plots. In addition, we extracted higher-level G-LiHT products of F-Cover, Tree Mean, Shrub Mean, and Pulse Density from the March and December 2017 datasets, which are provided at 13 m resolution but were filtered using a 3 × 3 averaging spatial window to get a 39 × 39 m average for each metric. To visualize the differences in the 3D point cloud from before (March) and after (December) Hurricane Irma passage, we conducted a comparative point cloud analysis for each of the 13 field plots located in the Shark River and Harney River study region. The comparative analysis indicates significant changes in vertical point cloud density distribution, as can be seen at an individual tree scale (Figure 3). Before Hurricane Irma, the point cloud had a high number of counts (very high density) in the upper canopy level, whereas after the hurricane the number of counts in the upper level (i.e., top canopy) was significantly reduced. The comparative analysis also shows a significant count increase near the ground and lower region of the point cloud (Figure 3). For most of the field plots, we also observed a decrease in the total amount of points calculated from the point cloud data after the storm, indicating a change in canopy structure, as observed in Table 2. In addition, a noticeable decrease in the tree height after Hurricane Irma can be detected based on changes in the point cloud from before and after Hurricane Irma comparison. Point cloud data taken from the same plot at SRS-6-50 (Shark River) can be seen in Figure 3c.

The nine-month time between the before (March 2017) and after (December 2017) G-LiHT data acquisitions imply the observed changes were induced by Hurricane Irma, or by other processes that occurred during this nine-month span. To evaluate if the observed changes were caused mainly by Hurricane Irma, we also calculated the observed G-LiHT changes around the scrub mangroves within Taylor Slough (Figure 1), which is the easternmost section of the Everglades mangrove forest and is located farthest away from the track of Hurricane Irma. Scrub mangroves in Taylor Slough did not have a significant impact on forest structure as a result of winds or a storm surge, due to their short stature and their far distance from the hurricane’s path [7]. We extracted data from six random locations within the mangroves of Taylor Slough that fell within the available G-LiHT data swaths and calculated the changes in different metrics (Table 1) before and after Hurricane Irma, which are provided in the Supplementary Material (Tables S4–S7). The comparison between the March (before) and December (after) data acquisitions revealed decreased values in F-Cover, Shrub Mean, Tree Means, and Pulse Density. However, the observed reductions in F-Cover and Tree Mean following the hurricane are significantly lower compared to F-Cover and Tree Mean change in the mangroves of the Shark and the Harney Rivers. The spatial pattern of significant F-Cover and Tree Mean changes in the western Everglades close to the hurricane track, and minor changes in the eastern Everglades farther away from the hurricane’s path suggests that the observed G-LiHT changes in our study area were induced mainly by Hurricane Irma.

### 5.2. Field-Based Estimates of AGN, WD, and AGTM

For each of the 13 field plots, mean WD, mean AGN, mean AGB, and mean AGTM was calculated using the appropriate line intercept method and allometric equation. Results of the mean field measurement for each of the 13 field plots can be seen in Table 3. We can see the mean WD value ranged from 3.0 Mg/ha to 157.6 Mg/ha and the highest value is seen in WSC-9-100. Distribution of WD varied, but in the Shark River, we see that values increase as one progresses from the edge of the forest (50 m) to the interior (350 m). For the Harney River, values seem to increase and then drop as one progresses from the forest edge to the interior. Mean AGN varies from 4 Mg/ha to 113.1, with the highest value seen in WSC-10-350. In the Shark River, AGN increases from the forest edge to the interior, but there is no strong pattern in the Harney River for AGN. Mean AGB values vary from 56.8 Mg/ha to 141.1 Mg/ha, with the highest values seen in SRS-6-350. In general, for both rivers, AGB seems to increase in volume and then drop, progressing from the forest edge to the interior. Values of AGTM vary from 92.60 Mg/ha to 187.4 Mg/ha, if we exclude the sampling error in SRS-6-100, with the highest volume seen in WSC-10-350. Within AGTM, we do not see a clear pattern of distribution within the two rivers. 

### 5.3. Regression Models

We systematically explored all models using changes in the four G-LiHT metrics (Table 1) to determine the best fit of the G-LiHT data to the measured values of AGN and WD. Changes in the G-LiHT metrics were defined as calculated values based on the pre-Irma (March 2017) minus the post-Irma (December 2017) acquisitions. An example of the fraction of first return intercepted by tree (F-Cover) metric change is presented in Table 4. Additional data representing change in Tree Mean, Shrub Mean, and Pulse Density are available in the Supplementary Material (Tables S1–S7). 

**Figure 4:**
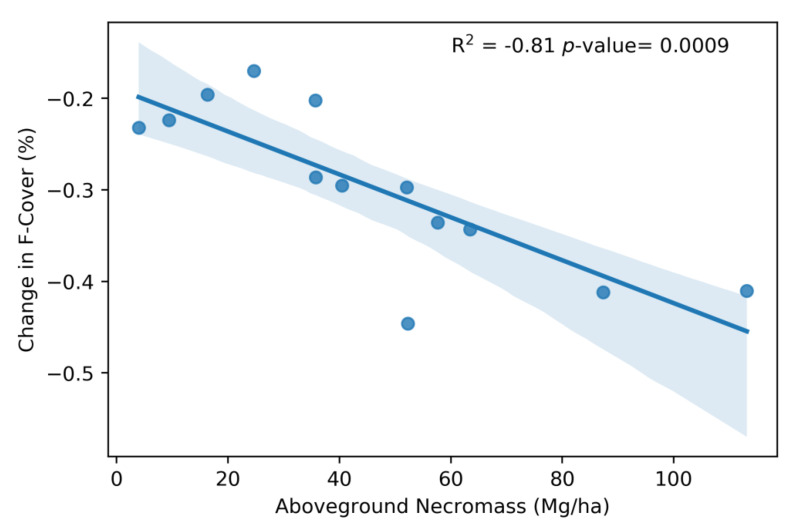
Regression model of AGN and change in F-Cover before and after Hurricane Irma where a regression of R^2^ = 0.81 was found using data from the 13 field plots. Shaded areas display the confidence intervals of the regression.

We ran multiple regression models for numerous combinations between AGN, WD, and lidar metrics in Table 1. However, the only statistically significant analysis that we report here is the regression for the AGN versus F-Cover (Figure 4). Additional regression plots can be found in the Supplementary Material (Figures S14 and S15). The systematic regression analyses between AGN, WD, and the changes in the four G-LiHT metrics shows that only one regression model, AGN vs. F-Cover, yielded a strong negative relationship (R^2^ = −0.81). All other regression models yielded poor positive or negative correlations (−0.3 < R^2^ < 0.3). These results indicate F-Cover is a good indicator for estimating AGN. However, they also suggest that none of the four G-LiHT metric changes are sensitive to the measured WD. 

For AGN-F-Cover, the regression equation is:

(3) AGN=a1+a2×ΔPFc

where *AGN* is the measured *AGN* value in each of the 13 plots, Δ*PFc* is the Percent Change in F-Cover, *a*1 is the intercept, and *a*2 is the slope of the regression. The best-fit analysis yielded the values of *a*1 = −34.11 and *a*2 = −304.18.

**Figure 5:**
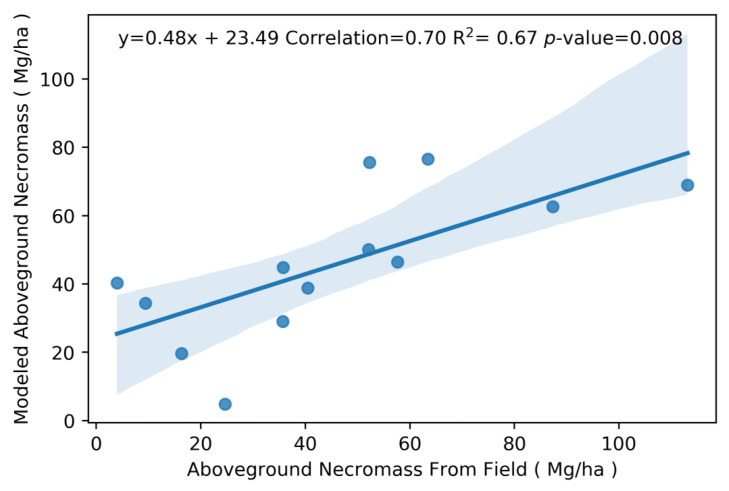
Relationship between measured AGN collected in the field versus modeled estimates of AGN using a regression analysis between field data and G-LiHT metrics. Shaded areas display the confidence intervals of the regression.

The percent changes in F-Cover were used as the independent variable in the standard regression model, whereas the AGN estimates calculated from the field vegetation surveys were used as the dependent variable. The linear regression model was calculated with an R^2^ = 0.81 and a *p*-value of 0.0009 for AGN. The root-mean-squared error (RMSE) calculated from the LOOCV algorithm resulted in an RMSE of 26.1 Mg/ha. We also calculated the residuals of the linear regression between AGN and F-Cover and found them to be on average +/− 14 Mg/ha. Modeled AGN versus AGN values collected in the field is shown to have a positive relationship with a R^2^ = 0.67 and *p*-value of 0.008 and a correlation score of 0.70 (Figure 5). 

### 5.4. Upscaling

**Figure 6:**
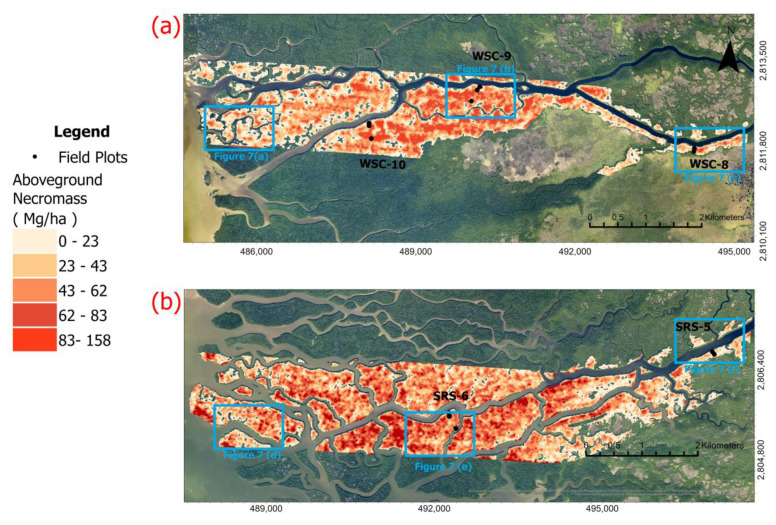
Upscaled distribution of modeled AGN estimates across the (**a**) Harney River and the (**b**) Shark River using the calculated linear regression equation. Blue squares are explained in Figure 7.

**Figure 7:**
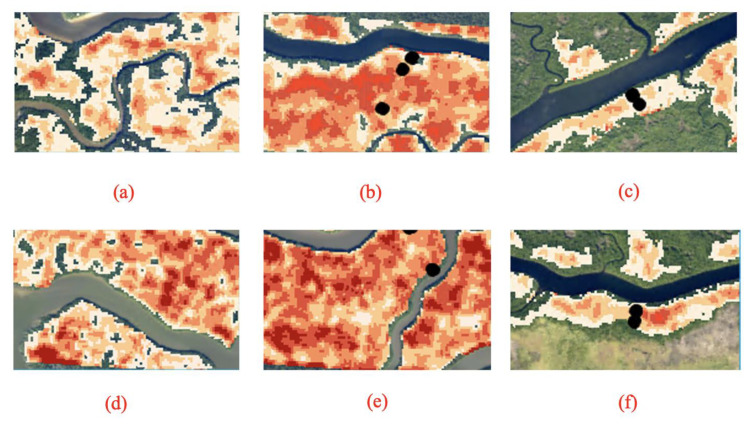
(**a**) Zoomed-in view of the mouth of the Harney River, (**b**) the downstream section (WSC-9) of the Harney River, and (**c**) the midstream section (WSC-8) of the Harney River. (**d**) shows a zoomed-in view of the mouth of the Shark River, (**e**) the downstream section (SRS-6) of the Shark River, and (**f**) the midstream section (site SRS-5) of the Shark River estuary.

Using the regression Equation (3), we calculated AGN values for the entire G-LiHT surveyed area extending along the Shark and Harney Rivers (Figure 6). AGN values were applied only to mangrove forest areas, which occupy the entire western section of the swaths and appear within narrow areas along tidal channels in the eastern section of the swathes. Along both swaths, the calculated AGN varies in the range of 0–140 Mg/ha. However, the distribution of higher AGN values varies between the two swaths. Along the Shark River, high AGN values occurred all along the swath, with higher concentration of AGN south of the Shark River near the mouth of the estuary. High AGN values occurred mostly in the midstream section of the Harney River, roughly 3–6 km west of the Gulf coast. The calculated AGN maps also show that along both swaths, high values occurred in patches 200–300 m wide, some elongated in the E-W direction and some bounded by water bodies (Figure 7).

**Figure 8:**
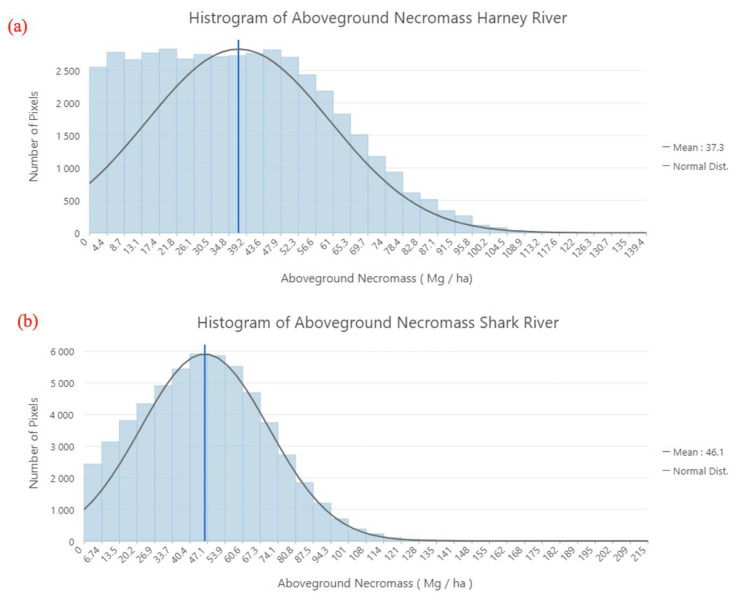
Histogram for the (**a**) Harney and (**b**) Shark River showing the number of pixels for each volume of aboveground necromass.

A comparison between the two AGN maps yielded the following main observations: (1) I Shark River swath has more damage than the Harney River swath based on the higher values of AGN observed along both swaths. The Shark River had an estimated total damage of 44,410 Mg and the Harney River had an estimated damage of 28,230 Mg. The mean volume of damage (i.e., AGN) for the Shark River was estimated to be 46.1 Mg/ha and 37.3 Mg/ha for the Harney River. (2) High concentrations of AGN in the Shark River are concentrated at the mouth of estuaries and in the downstream section, and the damage in the Harney River is most concentrated in the downstream section of the river. For both river segments, damage decreases progressing to the midstream section of each river and increases with distance from the mangrove edge to the interior of the forest along the main river channel (Figure 7). Both maps show that large areas of damage are primarily located in the interior of the mangrove forests and not along the edges of the riverbanks. Histograms for each river segment show the frequency of damage volume (Figure 8).

### 5.5. Ratio between AGN and AGTM

Using both AGN calculated in the field and modeled AGN, we took AGTM from the field and modeled AGTM from the CHM from G-LiHT to find the ratio of AGN to AGTM (Table 5). We found that for both the data from the field calculations and modeled data from G-LiHT, AGN makes up on average 29% of AGTB from at least a plot level. The ratio between AGN and AGTB also indicates the percentage of mortality for each field plot. This may vary spatially throughout our study region, but the analysis from our plots indicates a large loss of productive biomass and mortality of mangroves within our study region.

## 6. Discussion

First attempts to upscale AGN and WD using remote sensing data proved to be successful in modeling AGN, but unsuccessful for modeling WD. Our results show AGN was successfully upscaled due to high correlation between field AGN estimates and airborne observations, as well as low uncertainty values in modeled AGN. Due to poor correlation between field WD estimates and the airborne observations, we were not able to upscale WD estimates to a regional scale (i.e., estuary). This lack of relationship may be due to the small sample size of collected field data, or that the variation in WD on the forest floor post-disturbance could not be detected by G-LiHT.

**Figure 9:**
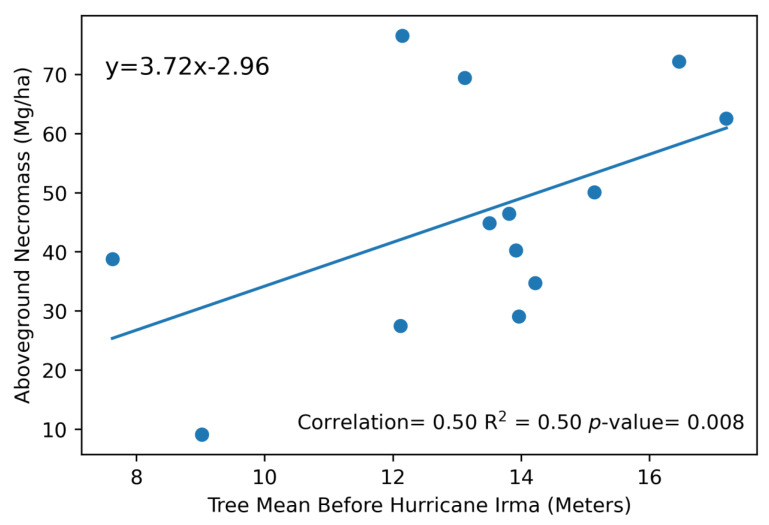
The linear relationship between Tree Mean before Hurricane Irma calculated from G-LiHT for each of the 13 field plots and modeled AGN for the same 13 field plots. Standard parameters of the linear model are included.

Since we were able to model AGN on a regional scale, we are able to assess damage distribution along each river. Using the calculated AGN model, we detect a high damage concentration in areas with tall trees (>10 m). We plotted Tree Mean and AGN from each of the 13 field plots (Figure 9) and found that there is a positive linear relationship between the two metrics, suggesting that tree height may play a role in mangrove vulnerability during hurricane impacts. Previous studies have shown that taller mangroves are more vulnerable to damage [8,9]. The high amounts of AGN positively correlating with tall mangrove tree heights also shows this relationship between mangrove height and vulnerability from hurricane disturbances.

Our observations indicate higher damage along the Shark River compared to the Harney River. We suggest that the higher damage along the Shark River could be the result of the interaction of the geomorphology of the coast, local microtopography, and storm physical properties [29]. The Shark River area, particularly the mouth, is comprised of several mangrove islands surrounded by more open water compared to that of the Harney River (Figure 1). This difference in surrounding vegetation could have played a role in the difference in damage between our two study areas. Indeed, the interaction of these factors has been attributed to control landscape variability in hurricane-induced sediment deposition (inland and laterally) across the FCE [7,29,30]. Mangroves have been proven to reduce wind speed to their surrounding environment for disturbances such as hurricanes [31]. Open water and warm water such as that found in South Florida can also lead to higher wind speeds affecting potential wind damage to the Shark River [32,33]. The path that Hurricane Irma took may also have affected the damage distribution between the Harney and Shark Rivers. However, both areas are geographically close to one another. Therefore, the distance difference between these two areas and the hurricane track may be minuscule and, hence, have a negligible impact on damage distribution between the two rivers. It is suggested though that the further the area is from the storm path, the more the energies from wind and storm surges decrease, which leads to decreased damage [12].

Since this is the first time AGN has been modeled for this region and at a large scale, we cannot compare modeled values of AGN to any previous hurricane storm or compare AGN to values found in other regions with disturbed mangroves. However, we can compare the percentage of damage within our study region to other regions and past storms. Damage to mangroves due to hurricane impacts can vary greatly due to various factors. For example, the region of Ten Thousand Islands, north of our study region, and the Florida Keys, south of our study region, were both hit directly by Hurricane Irma. Mangroves in the Ten Thousand Islands region showed a mortality of 11% and 19% in the Florida Keys [34]. Hurricane Wilma impacted our study region in 2005 as a Category 3 hurricane and caused a varied mortality of 3.5–15% along the mangroves of Shark River [35]. Our field plot data shows an average loss of 29% in biomass due to Hurricane Irma within the Shark and Harney rivers. This indicates that Hurricane Irma had a large impact within our study region when compared to two other areas impacted by Hurricane Irma and a past disturbance within our study region.

Mangroves provide many socio-ecological services, suggesting that hurricane-induced damages to mangroves may affect the benefits they provide at the regional scale in addition to both the ecological and monetary impacts. Areas with high damage that were detected by our AGN model have the potential to be hotspots of high nutrient and CO_2_ fluxes that may have negative impacts for this ecosystem in the future. Naturally, WD and other plant material will at some point start to decompose, and standing dead trees (AGN) will eventually break down and fall to the forest ground and continue to break down [36]. Fallen dead trees, along with any WD within the ground, will continue to decompose and leach both nutrients and gases such as CO_2_ into the environment [37,38].

## 7. Conclusions

By using high-resolution airborne lidar, we were able to successfully model and estimate hurricane-induced damages using AGN estimates for two river swaths of mangrove forests in the Everglades. Modeling AGN provided us an analysis on the distribution of damage Hurricane Irma caused on mangrove forests within our study region and suggests that spatial distance from the coast and forest edge, tree height, and surrounding landscape may play a role in the distribution of mangrove structural damage. Our results provide evidence that G-LiHT and airborne lidar are powerful tools in assessing large-scale hurricane disturbance and in improving our knowledge on how mangroves are affected by hurricanes on a canopy scale. However, our analysis also finds that G-LiHT alone is not sensitive enough to model finer-scale damage such as WD on the forest floor as we were unable to model WD using the proven G-LiHT data and metrics alone. Coupling G-LiHT with other remote sensing products may be beneficial for future research to attempt modeling volumes of WD in the future.

## Figures and Tables

**Table 1:** G-LiHT metrics relevant to this study and their definitions. Metrics were provided as 13 × 13 m^2^ gridded products.

Name	Description	Units
F-Cover	Fraction of first returns intercepted by trees	Fraction
Shrub Mean	Mean shrub returns heights Mean non-ground returns below 1.37 m	m
Tree Mean	Mean of tree returns heights Mean non-ground returns above 1.37 m	m
Pulse Density	Laser pulse density	Pulse/m^2^
Canopy Height Model	The height or residual distance between the ground pulse returns and the top of the pulse return labeled as trees (>1.37 m)	m

**Table 2:** Total pulse returns for each field plot before and after Hurricane Irma.

River	Site	Field Plot	Total Pulse Returns before Hurricane Irma	Total Pulse Returns after Hurricane Irma	Difference in Pulse Returns
Shark River	SRS-5	SRS-5-50	4850	3196	−1654
		SRS-5-100	4009	2678	−1331
	SRS-6	SRS-6-50	4792	4088	−704
		SRS-6-100	4637	4159	−478
		SRS-6-100 (2nd Plot)	4954	4102	−852
		SRS-6-350	2577	3200	623
Harney River	WSC-8	WSC-8-50	3043	3290	247
		WSC-8-100	1743	1789	46
	WSC-9	WSC-9-50	2840	1139	−1701
		WSC-9-100	3064	2695	−369
		WSC-9-350	2943	2514	−429
	WSC-10	WSC-10-50	1693	1938	245
		WSC-10-100	1605	1638	33
		WSC-10-350	2438	2290	−148

**Table 3:** Calculated mean WD, AGN, mean AGB, and AGTM for each field plot during the January 2018 in-field assessment from FCE LTER NSF RAPID assessment.

River	Site	Field Plot	Mean WD (Mg/ha)	Mean AGN (Mg/ha)	Mean AGB (Mg/ha)	AGTM (Mg/ha)
Shark River	SRS-5	SRS-5-50	30.7	16.4	140.7	157.0
		SRS-5-100	21.8	24.7	87.4	112.1
	SRS-6	SRS-6-50	2.6	52.3	119.6	172.0
		SRS-6-100	N/A	87.3	N/A *	N/A *
		SRS-6-100 (2nd Plot)	40.9	N/A	60.6	60.57
		SRS-6-350	52.3	4.00	141.1	145.1
Harney River	WSC-8	WSC-8-50	3.0	63.5	59.3	122.8
		WSC-8-100	9.8	40.5	65.3	105.7
	WSC-9	WSC-9-50	7.4	35.7	107.7	143.3
		WSC-9-100	157.6	52.1	99.63	151.8
		WSC-9-350	22.7	9.4	136.3	145.7
	WSC-10	WSC-10-50	23.7	57.6	99.6	157.2
		WSC-10-100	84.3	35.8	56.8	92.60
		WSC-10-350	19.7	113.1	74.2	187.3

* SRS-6-100 only had AGN value calculated due to error in sampling.

**Table 4:** Mean values of the F-Cover metrics derived from G-LiHT products for each of the 13 field plots before and after Hurricane Irma, and their differences.

River	Site	Field Plot	Mean Value of F-Cover (%) before Irma	Mean Value of F-Cover (%) after Irma	Change in F-Cover (%)	Percent Change in F-Cover
Shark River	SRS-5	SRS-5-50	96.7%	79.6%	−17.1%	−17.7%
		SRS-5-100	96.1%	83.8%	−12.3%	−12.8%
	SRS-6	SRS-6-50	93.8%	60.0%	−33.8%	−36.0%
		SRS-6-100	96.4%	65.7%	−30.7%	−31.8%
		SRS-6-100 (Plot 2)	96.4%	65.7%	−30.7%	−31.8%
		SRS-6-350	96.1%	72.6%	−23.5%	−24.5%
Harney River	WSC-8	WSC-8-50	96.7%	61.5%	−35.2%	−36.4%
		WSC-8-100	94.9%	72.2%	−22.7%	−23.9%
	WSC-9	WSC-9-50	96.9%	76.7%	−20.2%	−20.8%
		WSC-9-100	98.2%	71.0%	−27.2%	−287.7%
		WSC-9-350	97.8%	75.7%	−22.1%	−22.6%
	WSC-10	WSC-10-50	94.7%	69.6%	−25.1%	−26.5%
		WSC-10-100	95.0%	70.3%	−24.7%	−26.0%
		WSC-10-350	98.2%	64.9%	−33.2%	−33.9%

**Table 5:** Values of mean AGN, AGTM, and ratio between AGN and AGTM for both field data and modeled values calculated from G-LiHT CHM.

River	Site	Field Plot	Mean AGN (MG/ha)	AGTM (Mg/ha)	Mean AGN/AGTM (%)	Modeled AGN (Mg/ha)	Modeled AGTM (Mg/ha)	Modeled AGN/Modeled AGTM (%)
Shark River	SRS-5	SRS-5-50	16.4	157.0	10.5%	19.7	141.3	13.9%
		SRS-5-100	24.7	112.1	22.0%	4.8	118.3	4.1%
	SRS-6	SRS-6-50	52.3	172.0	30.4%	75.2	202.2	37.2%
		SRS-6-100	87.3	N/A	N/A	62.6	224.4	27.9%
		SRS-6-100 (Plot 2)	N/A	60.57	N/A	62.6	224.4	27.9%
		SRS-6-350	4.00	145.1	2.8%	40.3	138.9	29.0%
Harney River	WSC-8	WSC-8-50	63.5	122.8	51.7%	76.6	149.8	51.1%
		WSC-8-100	40.5	105.7	38.3%	38.8	78.5	49.4%
	WSC-9	WSC-9-50	35.7	143.3	24.9%	29.1	124.1	23.4%
		WSC-9-100	52.1	151.8	34.3%	50.1	174.0	28.8%
		WSC-9-350	9.4	145.7	6.5%	34.4	161.38	21.3%
	WSC-10	WSC-10-50	57.6	157.2	36.6%	46.5	164.7	28.2%
		WSC-10-100	35.8	92.60	38.7%	44.8	141.6	31.6%
		WSC-10-350	113.1	187.3	60.4%	67.0	164.2	40.8
Mean					29.8%			29.6%

## Data Availability

Data from NASA Goddard’s Lidar, Hyperspectral & Thermal Imager can be found openly at https://gliht.gsfc.nasa.gov/index.php?section=34 (accessed on 1 March 2022).

## Supplementary Materials

The following supporting information can be downloaded at: https://www.mdpi.com/article/10.3390/s23156669/s1. This material shows additional metric data from G-LiHT and point cloud models for each of the 13 field plots used in the study. Figure S1: Point cloud data for SRS-5-50; Figure S2: Point cloud data for SRS-5-100; Figure S3: Point cloud data for SRS-6-100; Figure S4: Point cloud data for SRS-6-100 (Second Plot); Figure S5: Point cloud data for SRS-6-350; Figure S6: Point cloud data for WSC-8-50; Figure S7: Point cloud data for WSC-8-100; Figure S8: Point cloud data for WSC-9-50; Figure S9: Point cloud data for WSC-9-100; Figure S10: Point cloud data for WSC-9-350; Figure S11: Point cloud data for WSC-10-50; Figure S12: Point cloud data for WSC-10-100; Figure S13: Point cloud data for WSC-10-350; Figure S14. Regression models for Wood Debris; Figure S15: Regression models for Aboveground Necromass; Table S1: Mean values for Tree Mean; Table S2: Mean values for Shrub Mean; Table S3: Mean values for Pulse Density; Table S4: Mean Values of F-Cover for Taylor Slough; Table S5: Mean Values of Tree Mean for Taylor Slough; Table S6: Mean Values of Shrub Mean for Taylor Slough; and Table S7: Mean Values of Pulse Density of Taylor Slough.
