# Supplementary material for: Estimating Structural Damage to Mangrove Forests Using Airborne Lidar Imagery: Case Study of Damage Induced by the 2017 Hurricane Irma to Mangroves in the Florida Everglades, USA
Source: Sensors (Basel). 2023 Jul 25;23(15):6669. doi: 10.3390/s23156669 (PMC10422621; doi:10.3390/s23156669)
Supplement: Supplementary file 1 [file sensors-23-06669-s001.zip › sensors-2410194-supplementary.pdf]

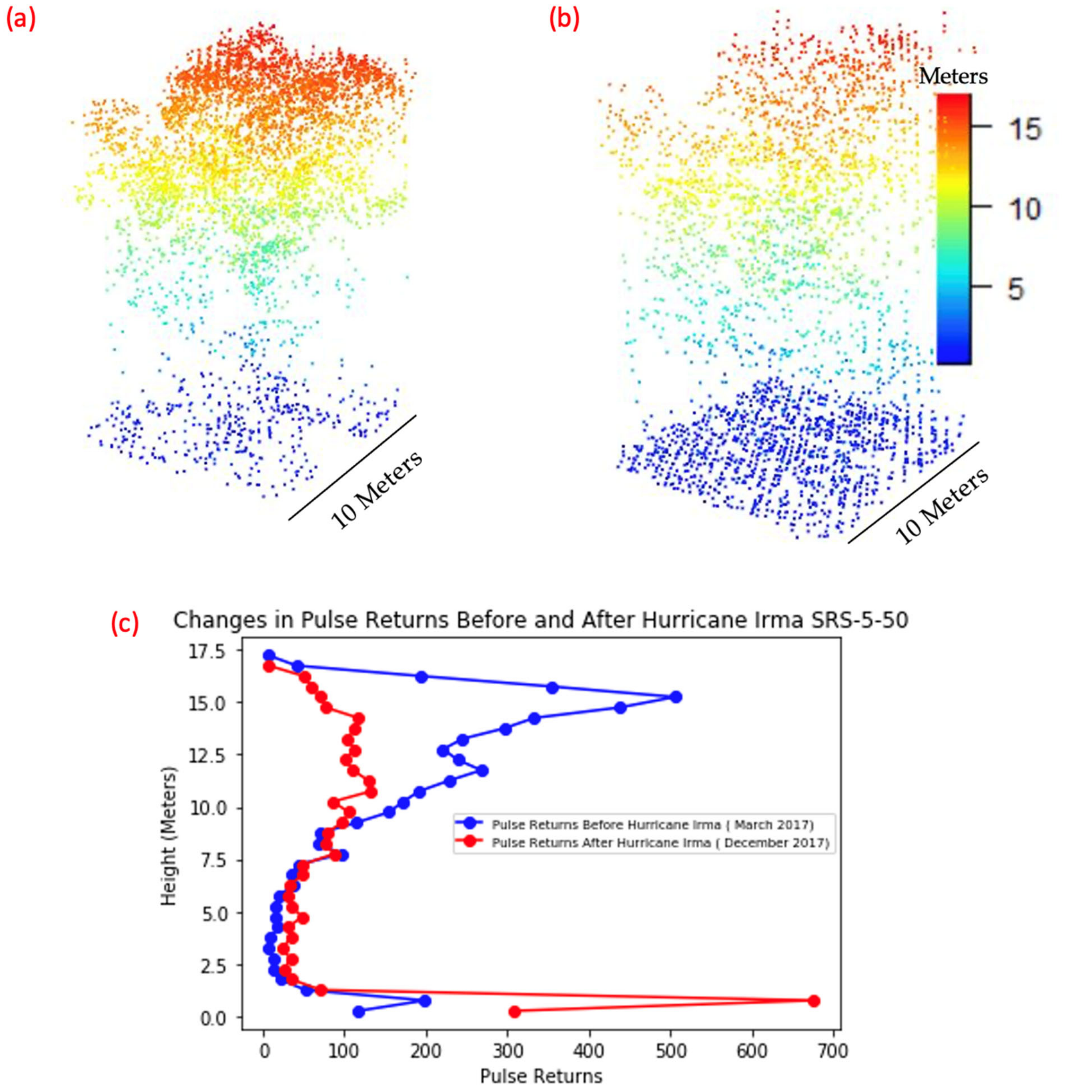

Figure S1. Point cloud variations of a single mangrove tree in plot SRS-5-50 in the Shark River study area before (a) and after (b) passage of Hurricane Irma. Distribution of pulse returns from point cloud data (c) at different height intervals from before Hurricane Irma versus after Hurricane Irma has been plotted. It can be noted that a high concentration of point returns is shown in higher elevation (canopy level) before Irma and at a lower elevation near soil surface after Irma.

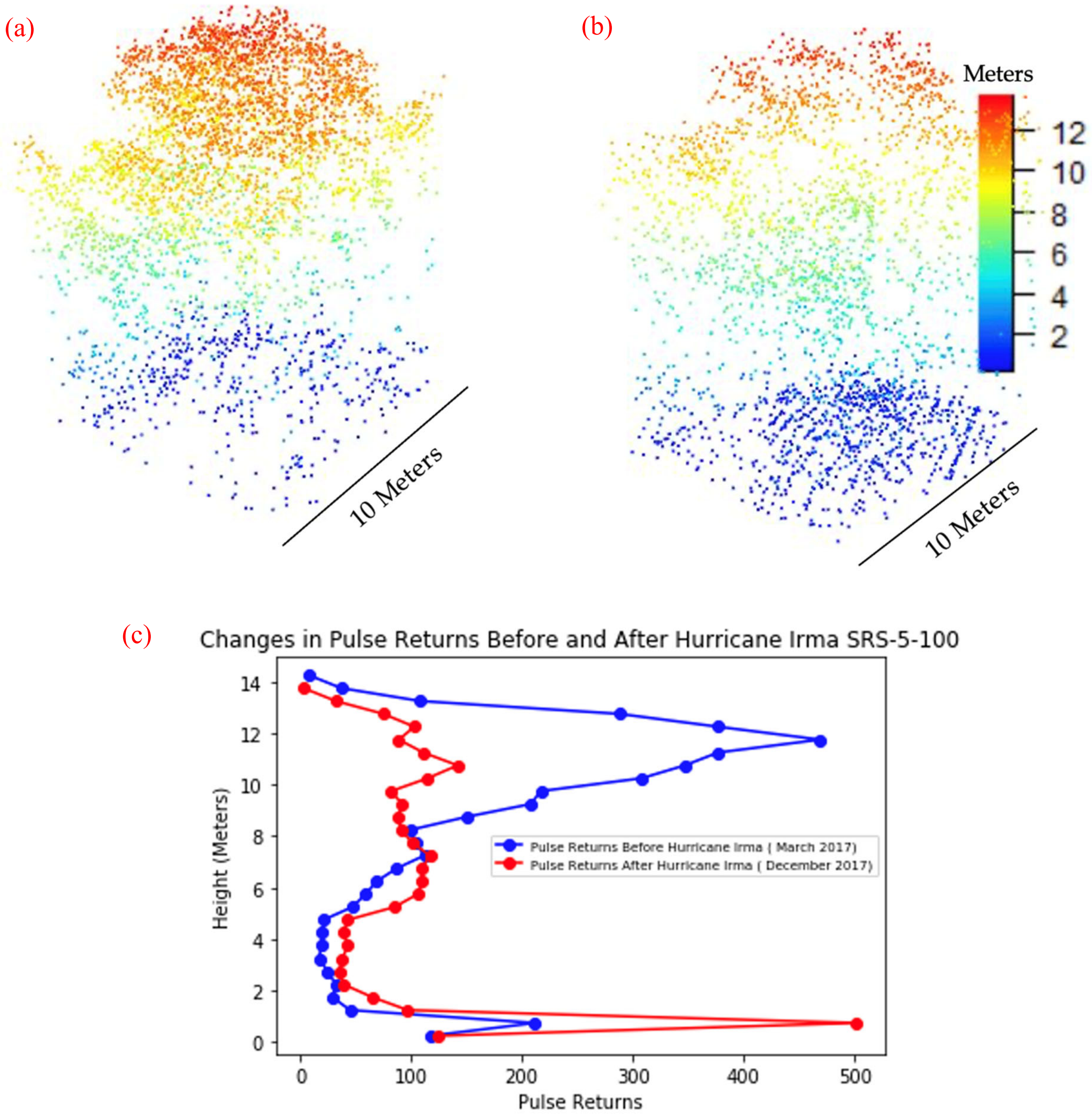

Figure S2. Point cloud variations of a single mangrove tree in plot SRS-5-100 in the Shark River area before (a) and after (b) passage of Hurricane Irma. Distribution of pulse returns from point cloud data (c) at different height intervals from before Hurricane Irma versus after Hurricane Irma has been plotted. It can be noted that a high concentration of point returns is shown in higher elevation (canopy level) before Irma and at a lower elevation near soil surface after Irma.

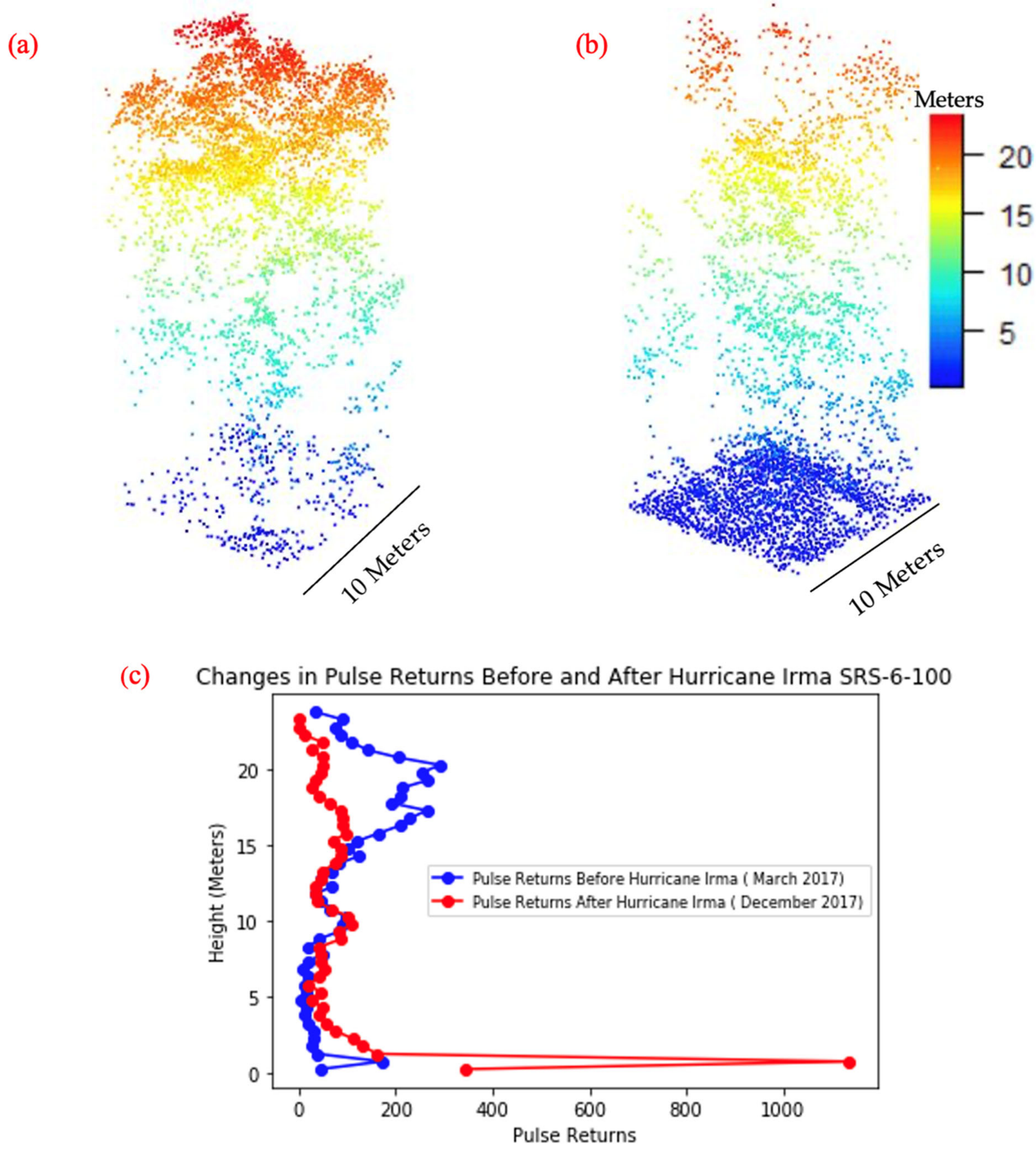

Figure S3. Point cloud variations of a single mangrove tree in plot SRS-6-100 in the Shark River study area before (a) and after (b) passage of Hurricane Irma. Distribution of pulse returns from point cloud data (c) at different height intervals from before Hurricane Irma versus after Hurricane Irma has been plotted. It can be noted that a high concentration of point returns is shown in higher elevation (canopy level) before Irma and at a lower elevation near soil surface after Irma.

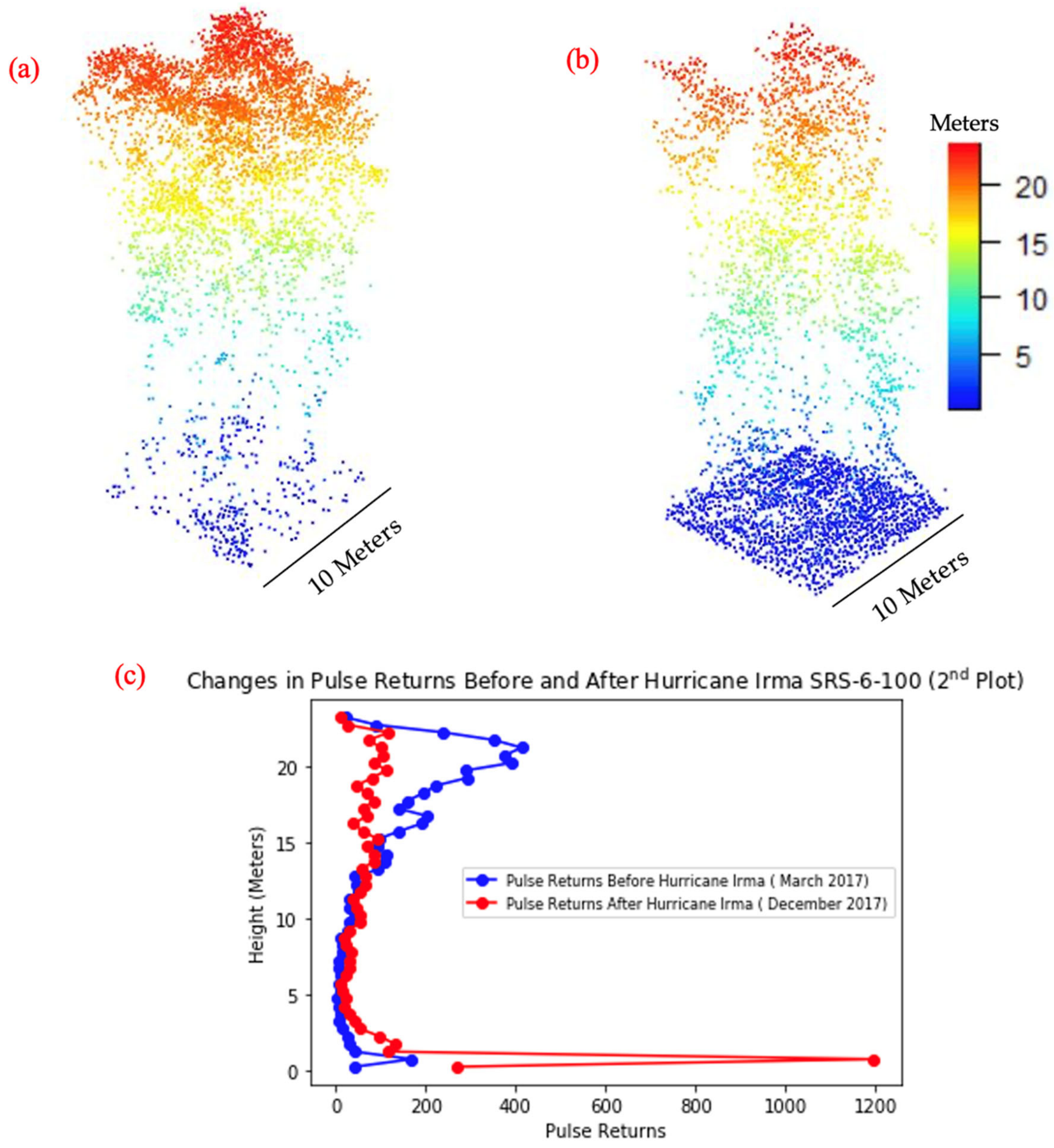

Figure S4. Point cloud variations of a single mangrove tree in plot SRS-6-100 (2nd Plot) in the Shark River study area before (a) and after (b) passage of Hurricane Irma. Distribution of pulse returns from point cloud data (c) at different height intervals from before Hurricane Irma versus after Hurricane Irma has been plotted. It can be noted that a high concentration of point returns is shown in higher elevation (canopy level) before Irma and at a lower elevation near soil surface after Irma.

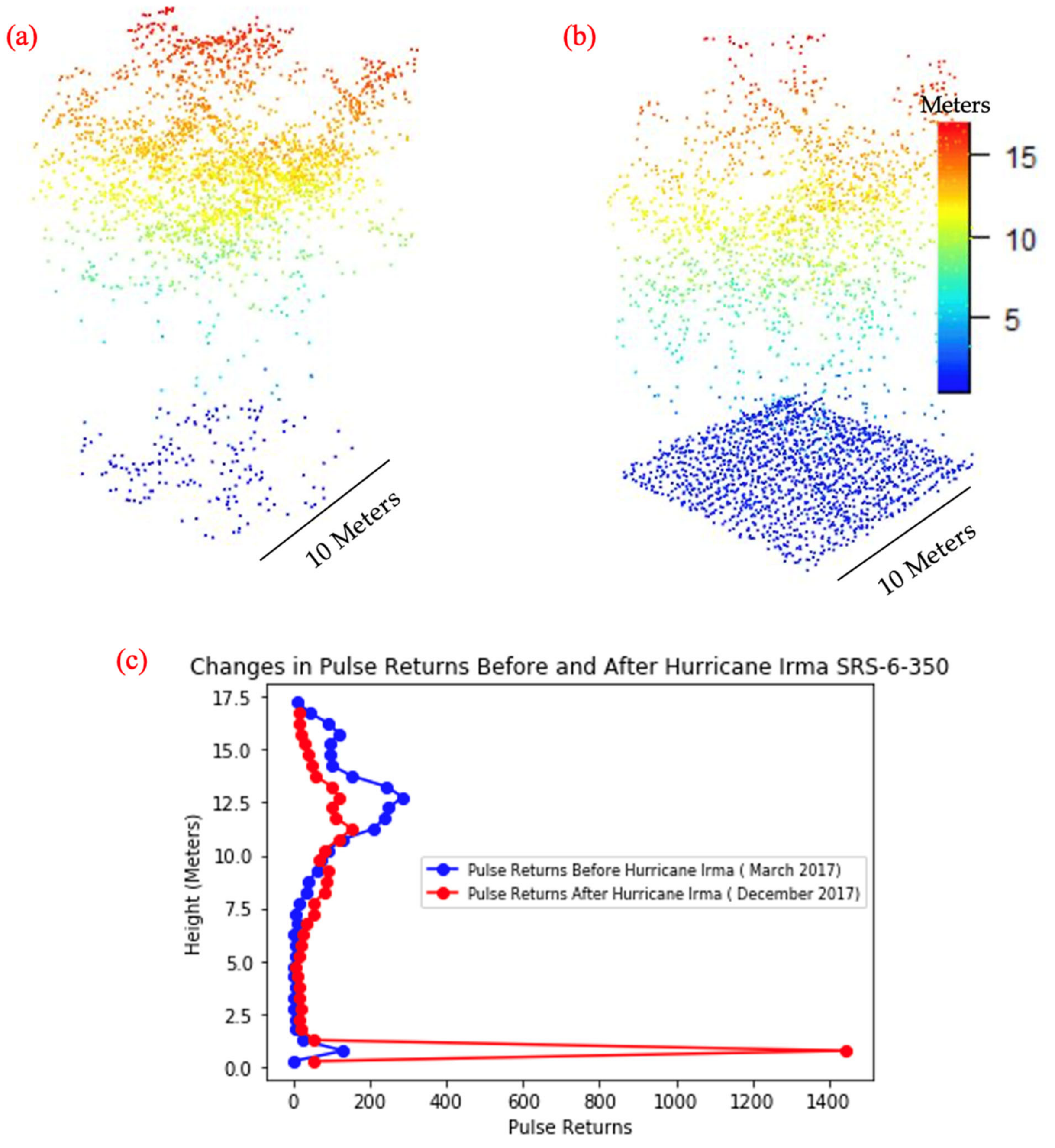

Figure S5. Point cloud variations of a single mangrove tree in plot SRS-6-350 in the Shark River study area before (a) and after (b) passage of Hurricane Irma. Distribution of pulse returns from point cloud data (c) at different height intervals from before Hurricane Irma versus after Hurricane Irma has been plotted. It can be noted that a high concentration of point returns is shown in higher elevation (canopy level) before Irma and at a lower elevation near soil surface after Irma.

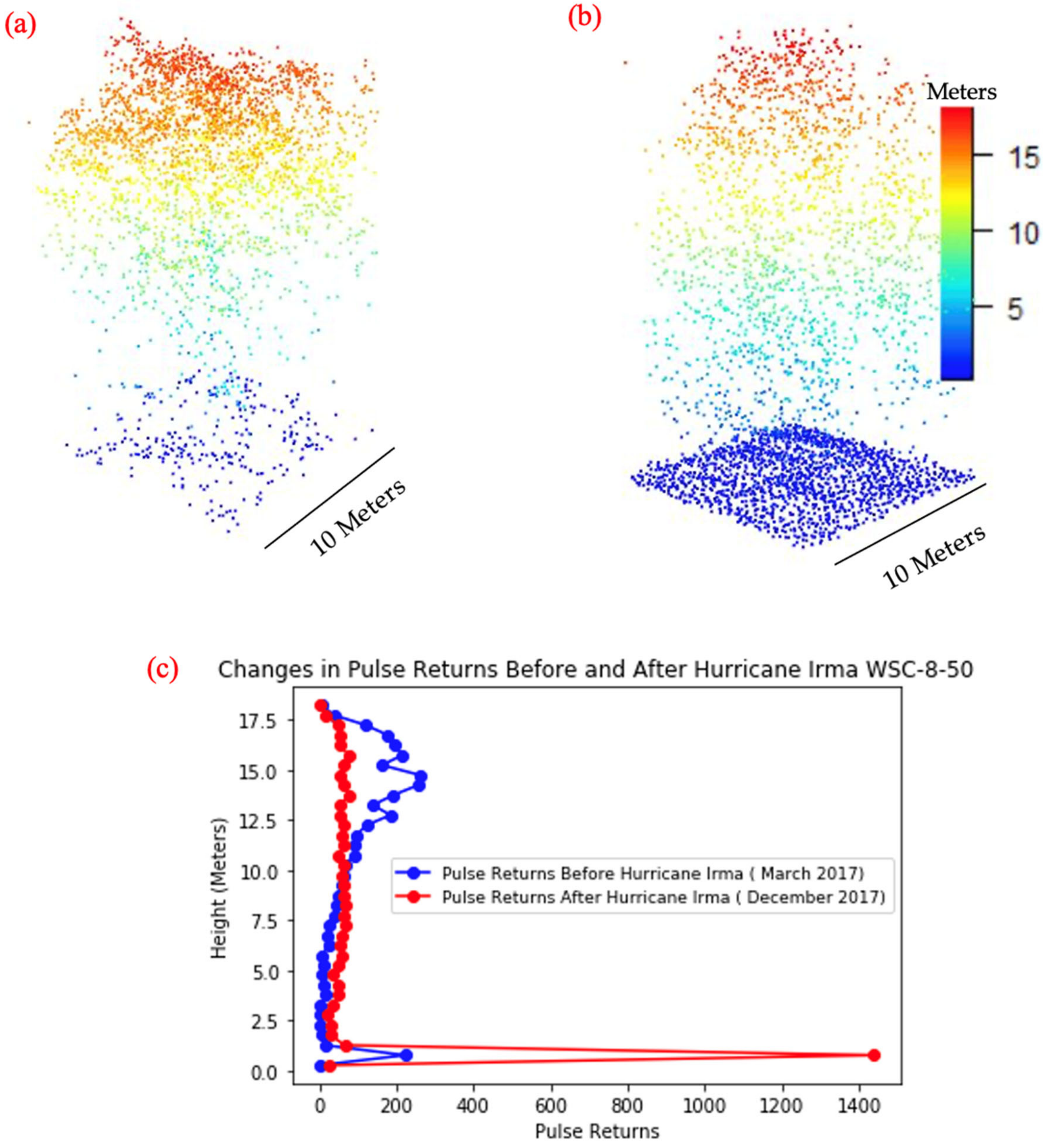

Figure S6. Point cloud variations of a single mangrove tree in plot WSC-8-50 in the Harney River study area before (a) and after (b) passage of Hurricane Irma. Distribution of pulse returns from point cloud data (c) at different height intervals from before Hurricane Irma versus after Hurricane Irma has been plotted. It can be noted that a high concentration of point returns is shown in higher elevation (canopy level) before Irma and at a lower elevation near soil surface after Irma.

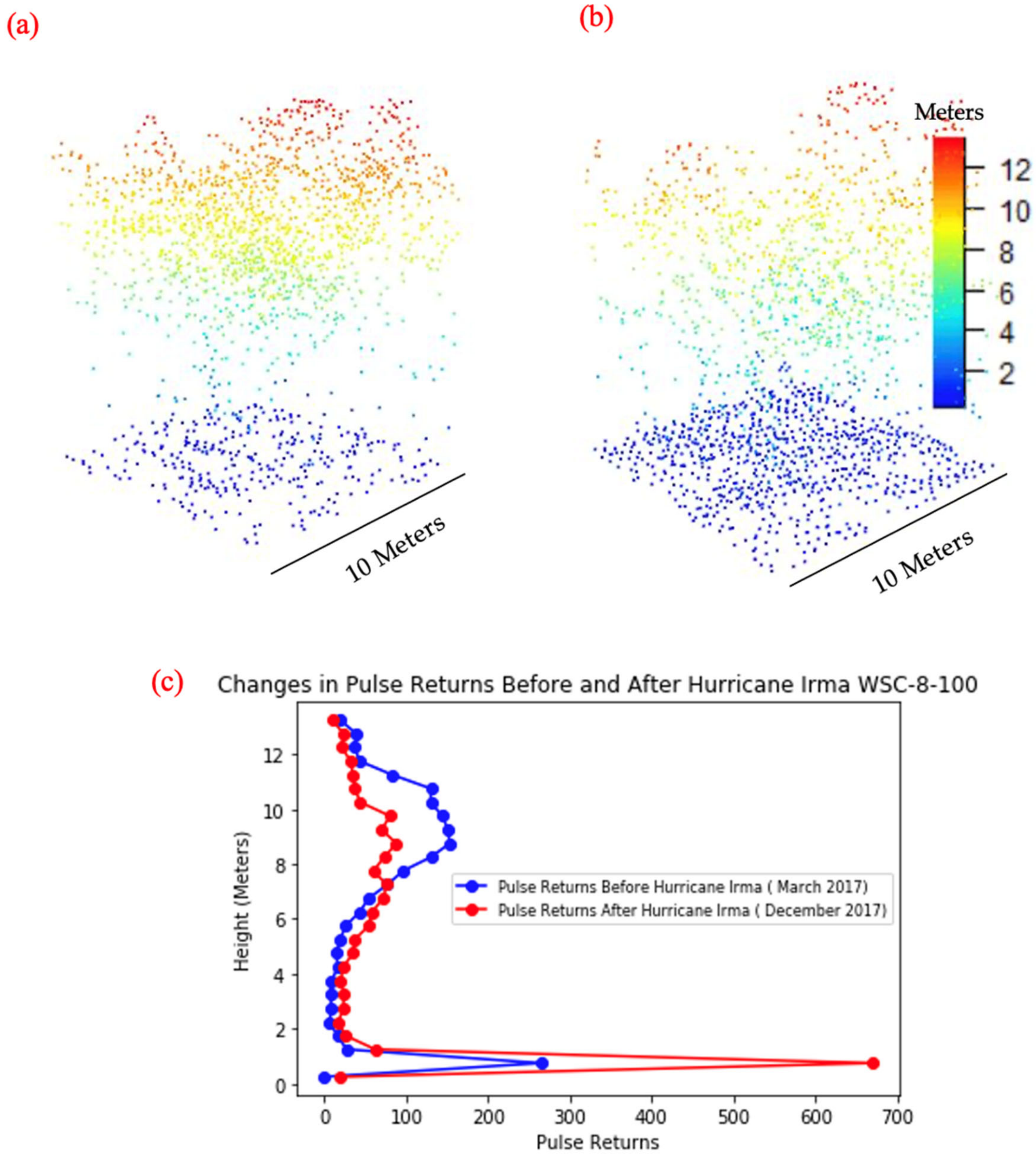

Figure S7. Point cloud variations of a single mangrove tree in plot WSC-8-100 in the Harney River study area before (a) and after (b) passage of Hurricane Irma. Distribution of pulse returns from point cloud data (c) at different height intervals from before Hurricane Irma versus after Hurricane Irma has been plotted. It can be noted that a high concentration of point returns is shown in higher elevation (canopy level) before Irma and at a lower elevation near soil surface after Irma.

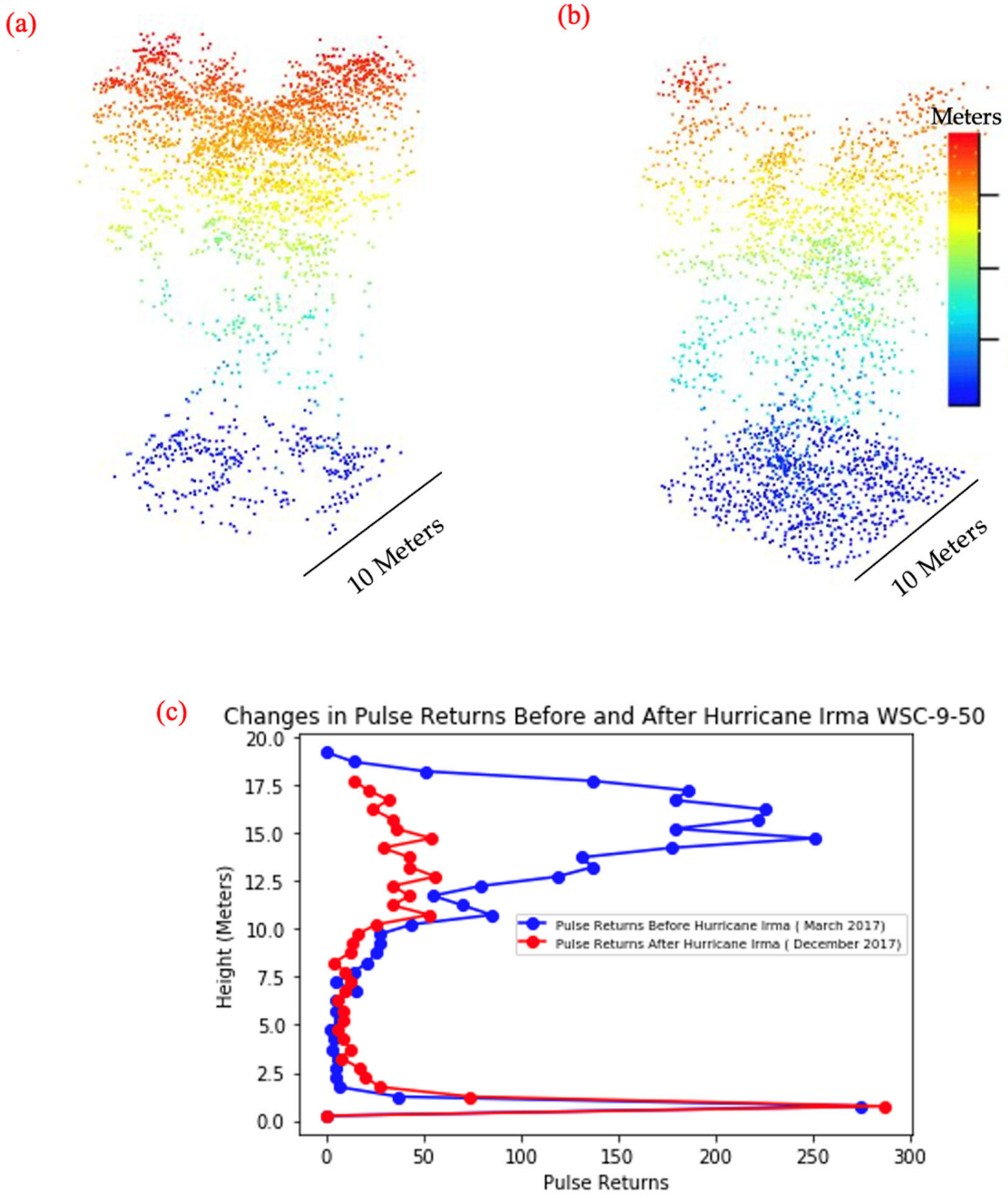

Figure S8. Point cloud variations of a single mangrove tree in plot WSC-9-50 in the Harney River study area before (a) and after (b) passage of Hurricane Irma. Distribution of pulse returns from point cloud data (c) at different height intervals from before Hurricane Irma versus after Hurricane Irma has been plotted. It can be noted that a high concentration of point returns is shown in higher elevation (canopy level) before Irma and at a lower elevation near soil surface after Irma.

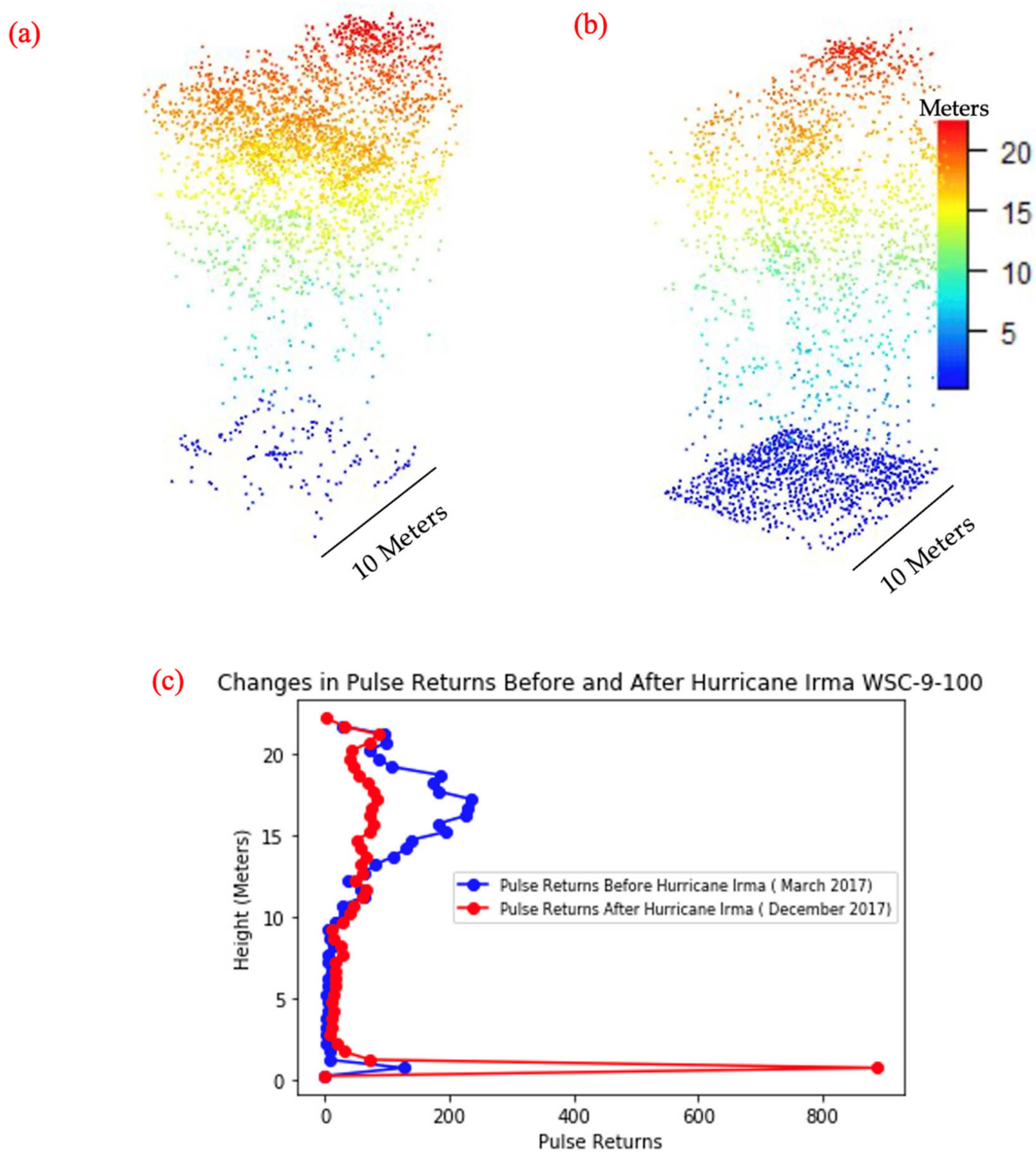

Figure S9. Point cloud variations of a single mangrove tree in plot WSC-9-100 in the Harney River study area before (a) and after (b) passage of Hurricane Irma. Distribution of pulse returns from point cloud data (c) at different height intervals from before Hurricane Irma versus after Hurricane Irma has been plotted. It can be noted that a high concentration of point returns is shown in higher elevation (canopy level) before Irma and at a lower elevation near soil surface after Irma.

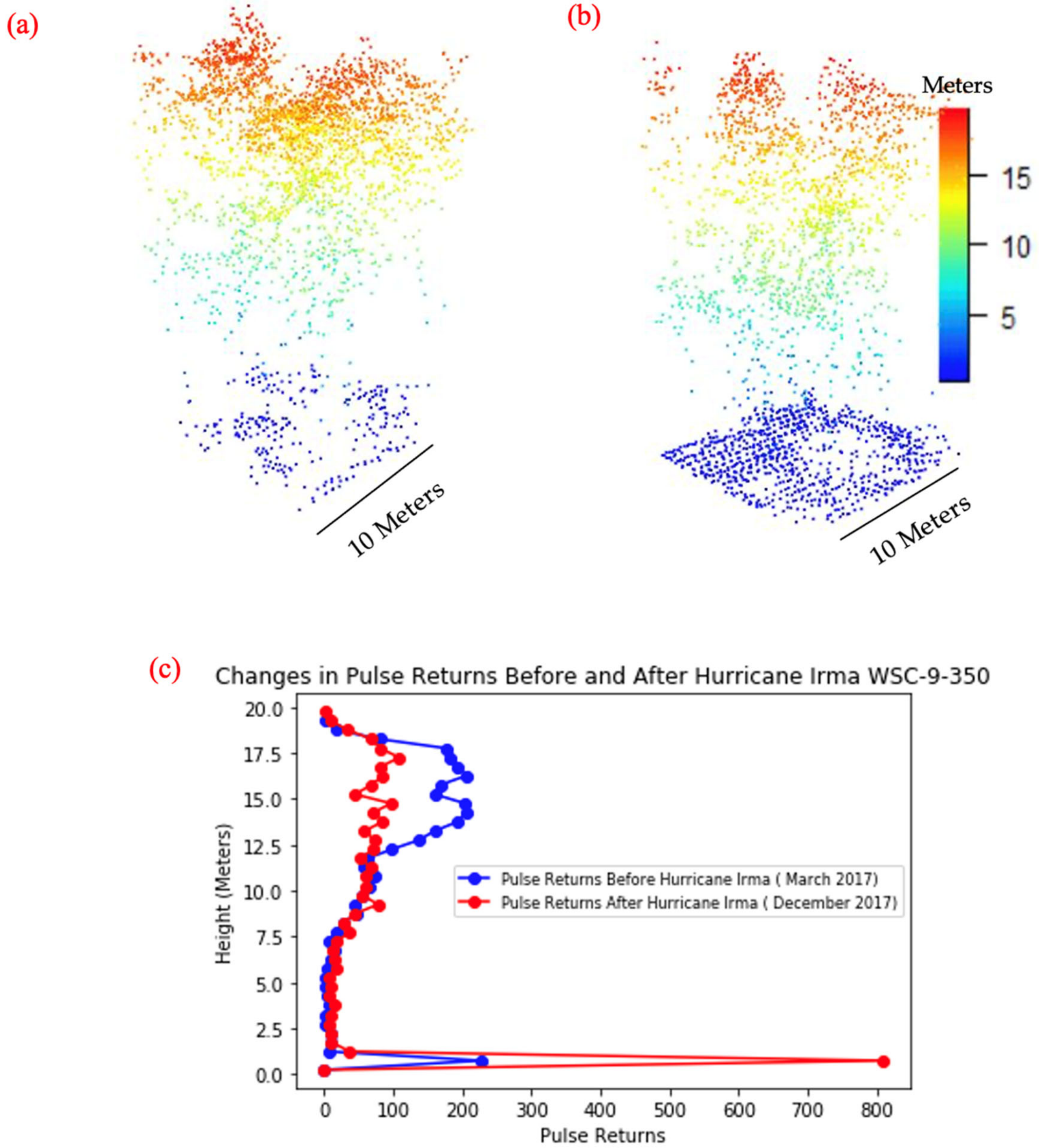

Figure S10. Point cloud variations of a single mangrove tree in plot WSC-9-350 in the Harney River study area before (a) and after (b) passage of Hurricane Irma. Distribution of pulse returns from point cloud data (c) at different height intervals from before Hurricane Irma versus after Hurricane Irma has been plotted. It can be noted that a high concentration of point returns is shown in higher elevation (canopy level) before Irma and at a lower elevation near soil surface after Irma.

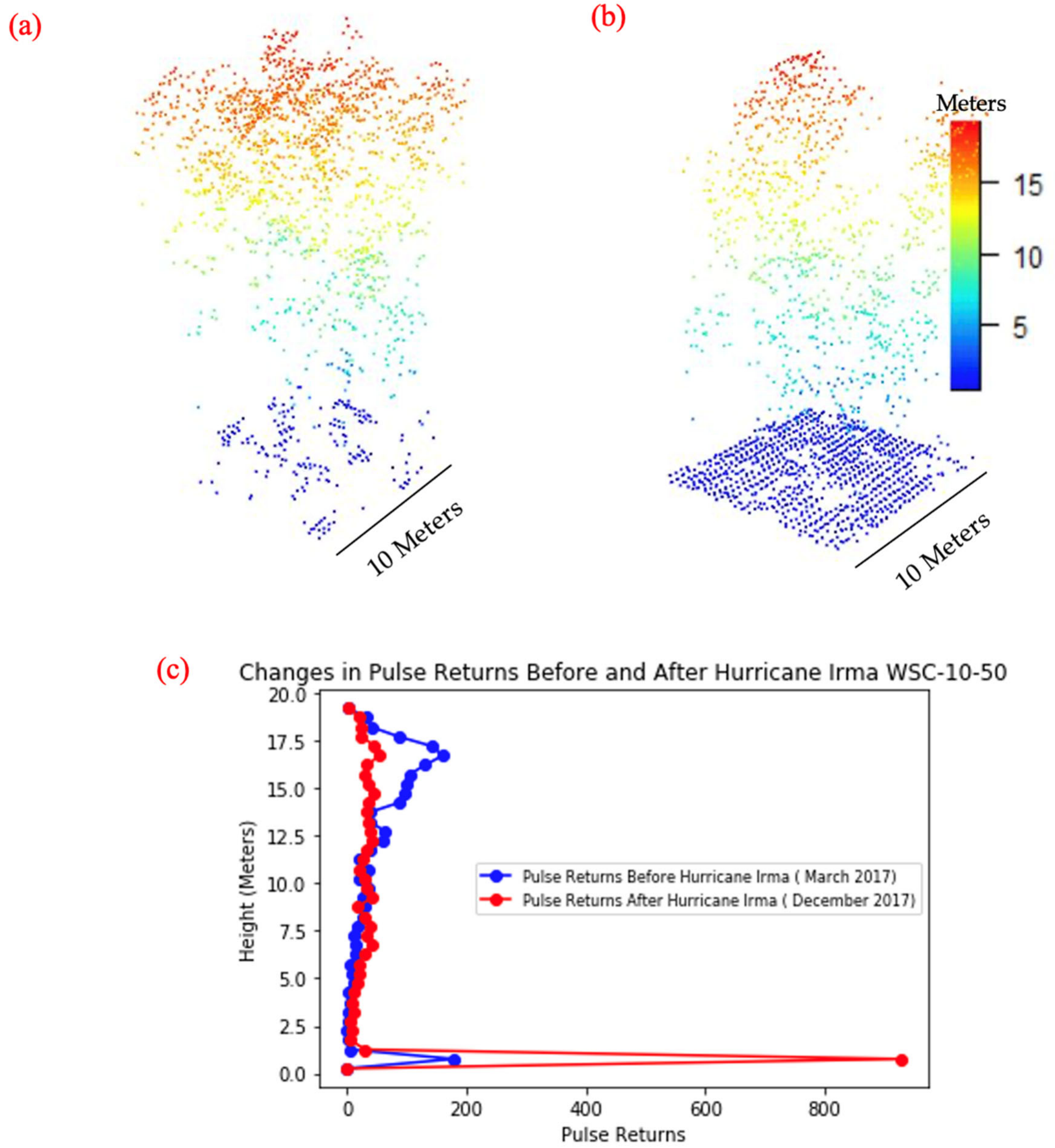

Figure S11. Point cloud variations of a single mangrove tree in plot WSC-10-50 in the Harney River study area before (a) and after (b) passage of Hurricane Irma. Distribution of pulse returns from point cloud data (c) at different height intervals from before Hurricane Irma versus after Hurricane Irma has been plotted. It can be noted that a high concentration of point returns is shown in higher elevation (canopy level) before Irma and at a lower elevation near soil surface after Irma.

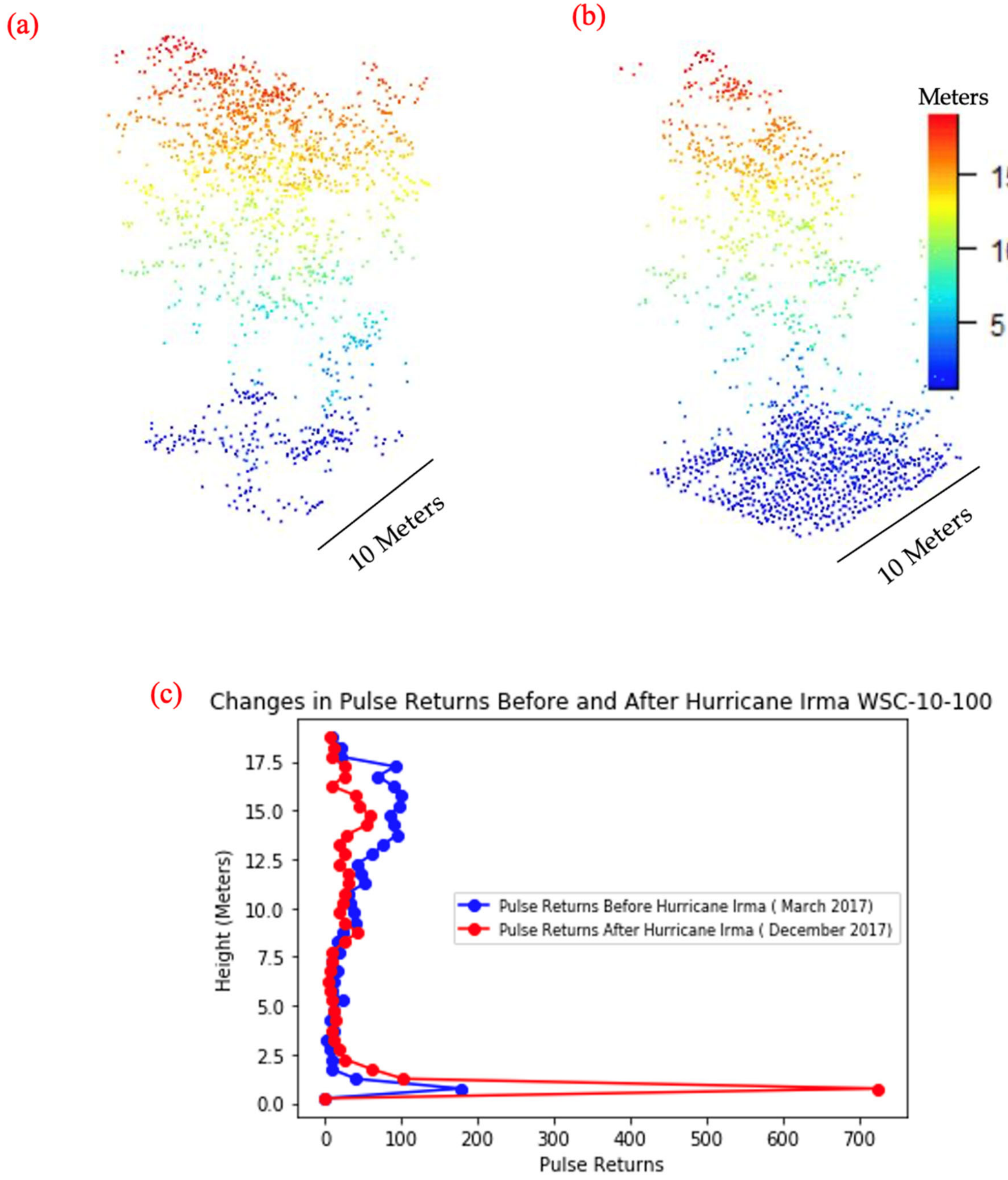

Figure S12. Point cloud variations of a single mangrove tree in plot WSC-10-100 in the Harney River study area before (a) and after (b) passage of Hurricane Irma. Distribution of pulse returns from point cloud data (c) at different height intervals from before Hurricane Irma versus after Hurricane Irma has been plotted. It can be noted that a high concentration of point returns is shown in higher elevation (canopy level) before Irma and at a lower elevation near soil surface after Irma.

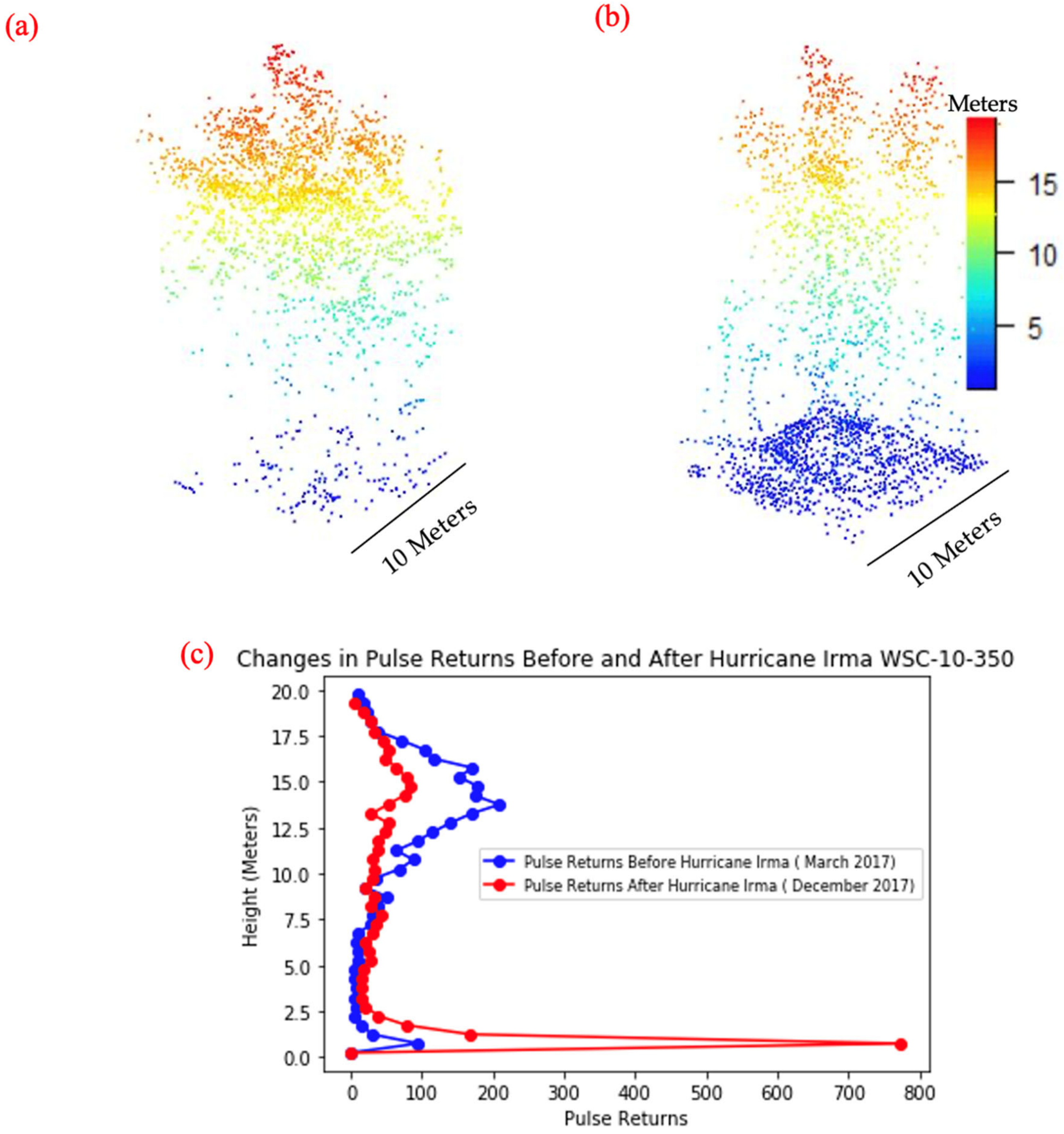

Figure S13. Point cloud variations of a single mangrove tree in plot WSC-10-350 in the Harney River study area before (a) and after (b) passage of Hurricane Irma. Distribution of pulse returns from point cloud data (c) at different height intervals from before Hurricane Irma versus after Hurricane Irma has been plotted. It can be noted that a high concentration of point returns is shown in higher elevation (canopy level) before Irma and at a lower elevation near soil surface after Irma.

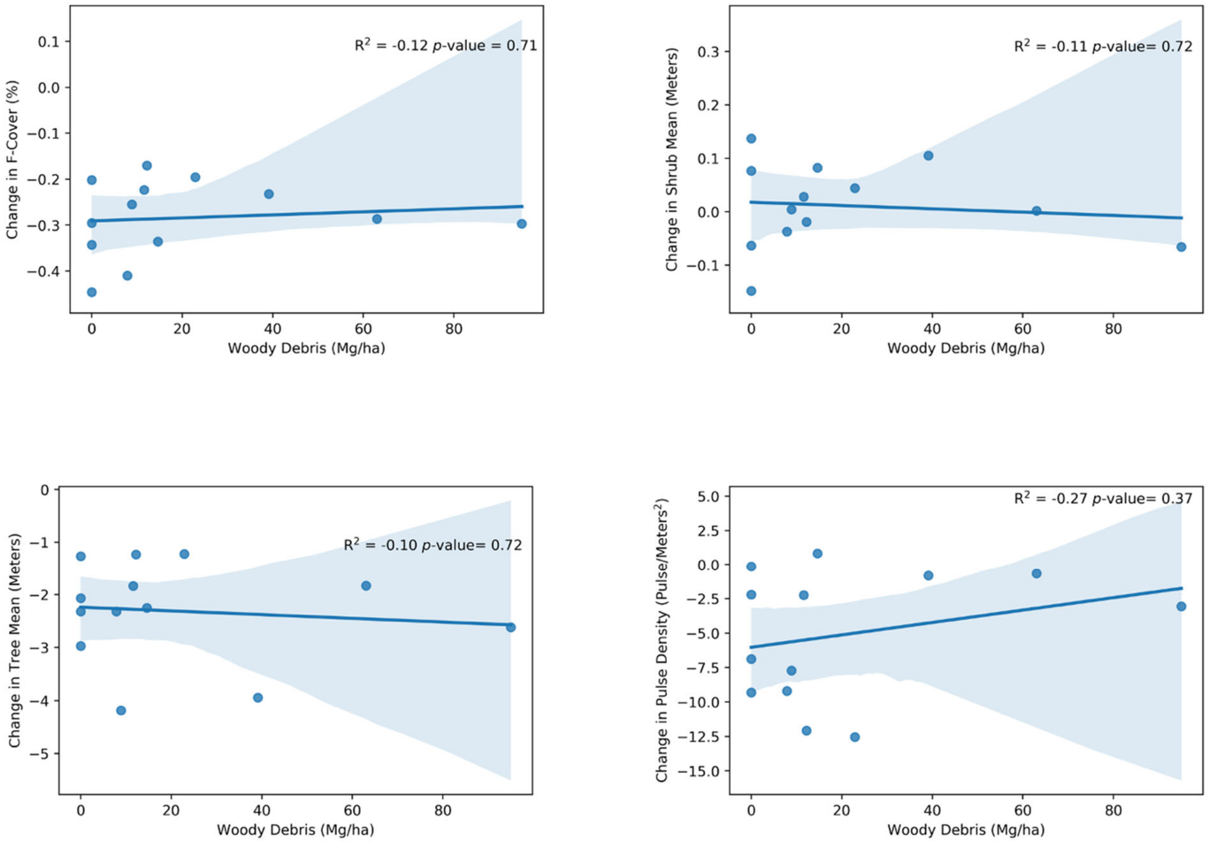

Figure S14. Regression models of the four G-LiHT metric changes (changes in F-Cover, Tree Mean, Shrub Mean, and Pulse Density) versus measure WD (Mg/ha) in the 13 in-field measured plots. Shaded areas display the confidence intervals of the regression

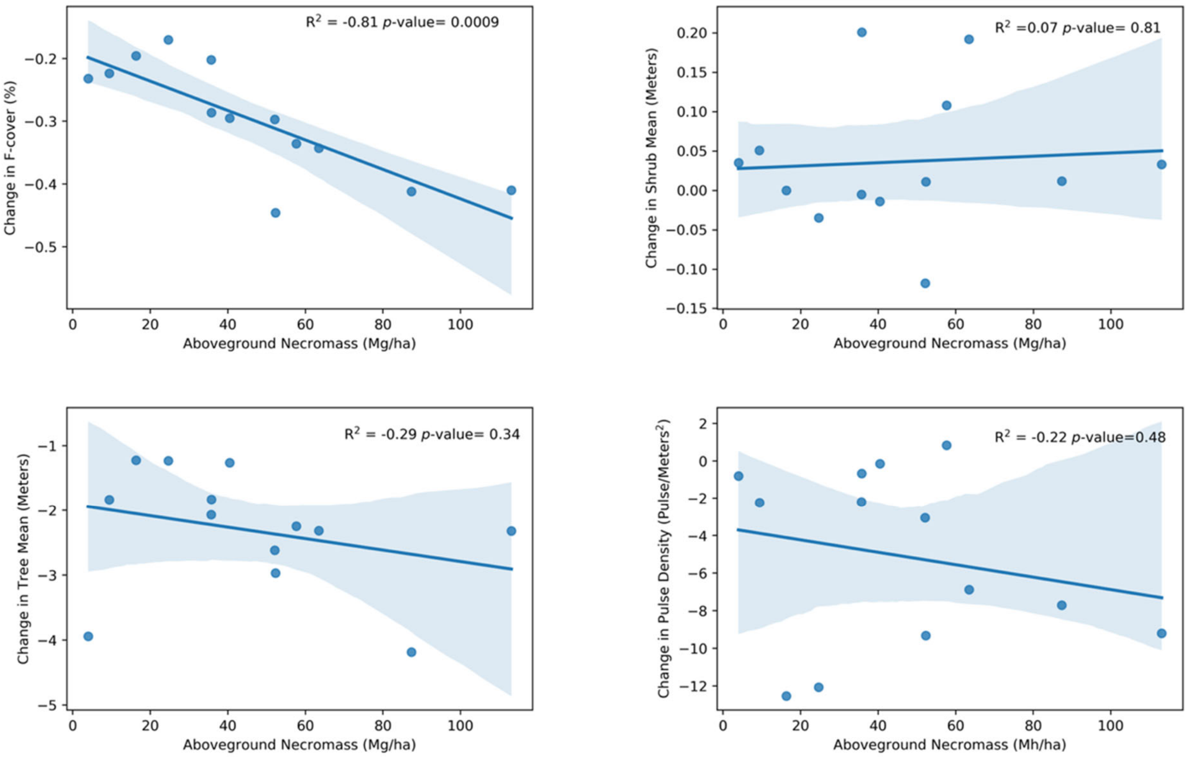

Figure S15. Regression models of the four G-LiHT metric changes (Changes in F-Cover, Tree Mean, Shrub Mean, and Pulse Density) versus measure AGN in the 13 in-field measured plots. Shaded areas display the confidence intervals of the regression.

Table S1. Mean values of the Tree Mean metrics derived from G-LiHT products for each of the 13 field plots before and after Hurricane Irma, and their differences.

River	Site	Field Plot	Tree Mean (Meters) Before Irma	Tree Mean (Meters)After Irma	Change in Tree Mean (Meters)	Percent Change in Tree Mean
Shark River	SRS-5	SRS-5-50	12.12	10.02	-2.10	-17%
		SRS-5-100	9.02	8.20	-0.82	-10%
	SRS-6	SRS-6-50	16.46	13.66	-2.8	-17%
		SRS-6-100	17.20	11.92	-5.28	-31%
		SRS-6-100 (2nd Plot)	17.20	13.76	-3.44	-20%
		SRS-6-350	13.91	11.79	-2.12	-15%
Harney River	WSC-8	WSC-8-50	12.14	9.15	-2.99	-25%
		WSC-8-100	7.62	6.57	-1.05	-14%
	WSC-9	WSC-9-50	13.35	11.84	-1.51	-11%
		WSC-9-100	15.20	13.38	-1.82	-12%
		WSC-9-350	14.21	12.49	-1.72	-12%
	WSC-10	WSC-10-50	13.82	11.27	-2.55	-18%
		WSC-10-100	13.50	12.20	-1.30	-10%
		WSC-10-350	13.12	11.18	-1.94	-15%

Table S2. Mean values of the Shrub Mean metrics derived from G-LiHT products for each of the 13 field plots before and after Hurricane Irma, and their differences.

River	Site	Field Plot	Shrub Mean (Meters) Before Irma	Shrub Mean (Meters)After Irma	Change in Shrub Mean (Meters)	Percent Change in Shrub Mean
Shark River	SRS-5	SRS-5-50	0.66	0.66	0	0%
		SRS-5-100	0.79	0.76	-0.03	-4%
	SRS-6	SRS-6-50	0.76	0.77	0.01	1%
		SRS-6-100	0.79	0.80	0.01	1%
		SRS-6-100 (2nd Plot)	0.79	0.77	-0.02	-3%
		SRS-6-350	0.61	0.65	0.04	7%
Harney River	WSC-8	WSC-8-50	0.69	0.88	0.19	26%
		WSC-8-100	0.78	0.77	-0.01	-1%
	WSC-9	WSC-9-50	0.61	0.60	-0.01	2%
		WSC-9-100	0.90	0.78	0.12	13%
		WSC-9-350	0.75	0.80	0.05	7%
	WSC-10	WSC-10-50	0.72	0.83	0.11	22%
		WSC-10-100	0.64	0.84	0.20	31%
		WSC-10-350	0.70	0.73	0.03	4%

Table S3. Mean values of the Pulse Density metrics derived from G-LiHT products for each of the 13 field plots before and after Hurricane Irma, and their differences.

River	Site	Field Plot	Mean Value of Pulse Density (Pulse/m^2) Before Irma	Mean Value of Pulse Density (Pulse/m^2) After Irma	Change in Pulse Density (Pulse/m^2)	Percent Change in Pulse Density
Shark River	SRS-5	SRS-5-50	31.98	19.44	-12.54	-39%
		SRS-5-100	31.20	19.12	-12.08	-39%
	SRS-6	SRS-6-50	23.44	14.11	-9.33	-40%
		SRS-6-100	31.96	24.25	-7.71	-21%
		SRS-6-100 (2nd Plot)	31.96	27.60	-4.36	-14%
		SRS-6-350	20.01	19.20	-0.81	-4%
Harney River	WSC-8	WSC-8-50	18.64	11.76	-6.88	-37%
		WSC-8-100	11.07	10.92	-0.15	-1%
	WSC-9	WSC-9-50	11.42	9.22	-2.2	-19%
		WSC-9-100	21.72	18.67	-3.05	-16%
		WSC-9-350	21.72	19.47	-2.25	-14%
	WSC-10	WSC-10-50	11.44	12.27	0.83	7%
		WSC-10-100	11.34	10.67	-0.67	-6%
		WSC-10-350	19.36	10.16	-9.2	-48%

Table S4. Mean values of the F-Cover metrics for 6 random point in the Taylor Slough

Field Plot	F-Cover (%) Before Irma	F-Cover (%) After Irma	Change in F-Cover (%)	Percent Change in F-Cover
1	49.0%	36.9%	-12.1%	-24.7%
2	73.9%	64.6%	-9.3%	-12.7%
3	26.5%	24.8%	-1.7%	-6.6%
4	33.2%	15.9%	-17.3%	-52.1%
5	12.8%	4.05%	-8.8%	-68.7%
6	27.1%	16.5%	-10.6%	-39.2%

Table S5. Mean values of the Tree Height metrics for 6 random point in the Taylor Slough

Field Plot	Mean Tree Height (m)Before Irma	Mean Tree Height (m)After Irma	Change in mean Tree Height (m)	Percent Change in Mean Tree Height
1	2.0	2.0	0	0%
2	2.7	2.5	-0.2	-7.4%
3	2.8	2.6	-0.2	-7.1%
4	1.7	1.7	0	-0%
5	1.8	2.0	0.2	11.1%
6	1.9	1.9	0%	-0%

Table S6. Mean values of the Pulse Density metrics for 6 random point in the Taylor Slough

Field Plot	Mean Pulse density (Pulse/m²) Before Irma	Mean Pulse Density (Pulse/m²) After Irma	Change in mean Pulse Density (Pulse/m²)	Percent Change in Pulse Density
1	8.4	6.3	-2.1	-25.2%
2	9.7	11.8	2.1	21.0%
3	12.3	12.5	0.2	1.3%
4	10.3	11.4	1.1	10.4%
5	6.9	5.8	1.1	-16.6%
6	9.1	9.0	0.1	-0.9%

Table S7. Mean values of the Shrub Mean metrics for 6 random point in the Taylor Slough

**Field Plot**	**Mean *Shrub Mean* (m)Before Irma**	**Mean Shrub Mean (m)After Irma**	**Change in mean Shrub Mea*n***	**Percent Change in Mean Shrub Mean**
1	0.94	0.77	-0.17	18%
2	0.75	0.78	-0.03	4%
3	1.0	1.0	0	0%
4	1.0	0.76	0.24	24%
5	0.86	0.58	0.28	33%
6	0.89	0.70	0.19	21%
